# Matrix-free human lung organoids derived from induced pluripotent stem cells to model lung injury

**DOI:** 10.1186/s13287-024-04106-3

**Published:** 2024-12-18

**Authors:** Bettina Budeus, Chiara Kroepel, Lisa Marie Stasch, Diana Klein

**Affiliations:** Institute for Cell Biology (Cancer Research), University Hospital Essen, University of Duisburg-Essen, Essen, Germany

**Keywords:** Lung organoid, iPSC, Lung injury, Disease modeling, Genotoxic stress, Single cell RNAseq, Radiation

## Abstract

**Background:**

Organoids, as near-physiological 3D culture systems, offer new opportunities to study the pathogenesis of various organs in mimicking the cellular complexity and functionality of human organs.

**Method:**

Here we used a quite simple and very practicable method to successfully generate induced pluripotent stem cell (iPSC)-derived human lung organoids (LuOrg) in a matrix-free manner as an alternative to the widely used preclinical mouse models in order to investigate normal lung damage in detail and as close as possible to the patient. We performed detailed morphological and molecular analyses, including bulk and single cell RNA sequencing, of generated lung organoids and evaluated the quality and robustness of our model as a potential in vitro platform for lung diseases, namely radiation-induced lung injury.

**Results:**

A matrix-free method for differentiation of iPSCs can be used to obtain lung organoids that morphologically reflect the target tissue of the human lung very well, especially with regard to the cellular composition. The different cellular fates were investigated following the genotoxic stress induced by radiation and revealed further insights in the radiation-sensitivity of the different lung cells. Finally, we provide cellular gene sets found to be induced in the different lung organoid cellular subsets after irradiation, which could be used as additional RT response and particularly senescence gene sets in future studies.

**Conclusion:**

By establishing these free-floating LuOrgs for the investigation of cancer therapeutic approaches as a new and patient-oriented in vitro platform particularly in experimental radiooncology, not only a reduction in the number of experimental animals, but also an adequately and meaningfully replacement of corresponding animal experiments can be achieved.

**Supplementary Information:**

The online version contains supplementary material available at 10.1186/s13287-024-04106-3.

## Graphical abstract

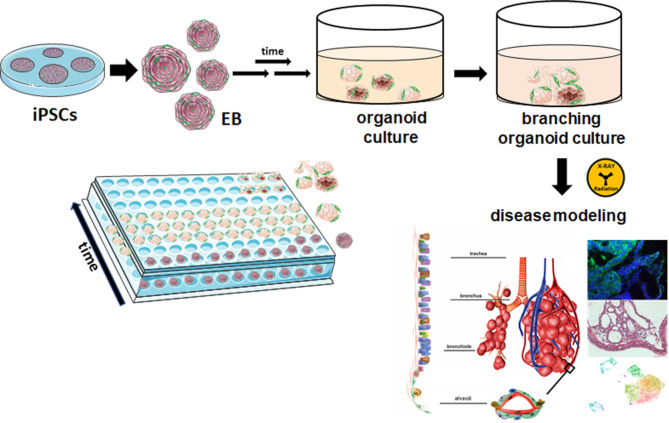

## Background

A central component of drug research was and is the use of pathological cells as well as normal tissue cells and preclinical mouse models, including patient-derived xenografts. Since the advent of organoid technology, numerous tissues can now be cultured in vitro as organoids, which are obtained directly from the tissue of an individual and represent an attractive ex vivo platform for studying patient-related cell biology [1, 2], including genetic profiles and therapy sensitivities [3, 4]. For example, it has already been shown that drug response in patients could be matched to that of organoid cultures by up to 90% [5]. Organoids are a valuable alternative to animal models to recapitulate cell biology as well as the complex interactions between different pathological (mostly neoplastic) cells, with the formed extracellular matrix and even with co-cultured stromal cells [6, 7]. Here, the use of disease-specific organoids has facilitated the analysis of underlying molecular mechanisms and successfully paved the way to personalized medicine by identifying potentially new biomarkers and developing patient-specific platforms for toxicological studies.

Lung organoids can thus be regarded as state-of-the-art platforms for modeling lung diseases in vitro in a patient-like manner and also facilitate the testing of potential therapeutic substances for the development of new drugs [3, 8]. A disadvantage of using established lung epithelial cell lines or primary cell cultures generated from lung biopsy material is the rather high donor variability. At the same time, however, only a few human tissues are accessible for investigation. The establishment of organoids typical of the tissue of origin, which were generated from induced pluripotent stem cells (iPSCs), has remedied this problem. In this ethically acceptable variant of pluripotent cells, specific gene sequences (Oct 3/4, Sox2, c-Myc and Klf4) are transferred into the target cells using viral or non-viral gene transfer in order to then reprogram these cells into the undifferentiated embryonic stem cell stage [9]. Since iPSCs were first obtained, impressive progress has been made in ‘engineering’ desired target cells from these pluripotent stem cells, and these iPSCs are now also commercially available. Compared to the animal models used to date, iPSC-derived lung organoids have the advantages that they (i) are derived from human cells, (ii) all have the same genetic background and (iii) can be produced in larger quantities (especially for (patho)-physiology modeling and therapeutic testing) [4, 10].

## Materials and methods

### Generation and culture of lung organoids (LuOrgs)

Two commercially available human iPSC lines were used. The SCTi003 iPSC cell line (iPSC #1) derived from healthy female donor peripheral blood mononuclear cells (PBMCs) was purchased from StemCell Technologies (Vancouver, BC, Canada), and the iPS01 iPSC cell line (iPSC #2) derived from human foreskin fibroblasts by retroviral expression of OCT4, SOX2, KLF4, and c-MYC genes from ALSTEM Inc (Richmond, CA, USA). Human iPSCs were maintained on Vitronectin XF™-coated culture dishes with mTeSR™1 (StemCell Technologies (Vancouver, BC, Canada) according to the manufacture’s protocols (/www.stemcell.com/guide-to-passaging-human-pluripotent-stem-cells-using-mtesr1.html). The cells were cultured under standard cell culture conditions at 37 °C and 5% CO_2_. All cells were routinely tested for mycoplasma contamination (every month). For LuOrg generation, iPSCs were harvested using an enzyme-free cell dissociation reagent (GCDR, #100–0485; StemCell Technologies), counted, and seeded into ultra-low attachment BIOFLOAT 96-well plate (#83.3925.400; SARSTEDT, Nümbrecht, Germany) in mTeSR1 medium (#15883465; STEMCELL Technologies, Vancouver, Canada) with 2500 cells per 100 µl per well. After 4–5 days, 100 µl per well of Branching Lung Organoid Medium (BLO-Medium) was added to generated embryoid bodies. After additional 4-5d days, a complete media change was performed and structures were cultured for 28–52 days with complete media changes every other day. The BLO-Medium) consists of the following components as previously reported [8]: DMEM F12 with GlutaMAX™ Supplement (#10565018;GIBCO/Invitrogen/Thermo Fisher Scientific, Carlsbad, CA, USA), 1× N-2, 1× B27, 1× penicillin–streptomycin (5,000 U/mL), 0.4% (vol/vol) BSA, 0.4 µM monothioglycerol, 50 µg/mL ascorbic acid, 10 ng/mL KGF/FGF7, 10 ng/mL FGF10; 50 nM ATRA, 3 µM CHIR-99,021. All used materials including source and identifier are additionally listed in Table S2 (key resources table).

### Histology and immunofluorescence

Immunohistochemistry (IHC) and immunofluorescence staining were performed on formalin-fixed and paraffin-embedded LuOrgs as previously described [11]. At indicated time points, LuOrgs were treated with 4% para-formaldehyde/ phosphate buffered saline (PBS) for 30 min, and subsequently subjected for paraffin-embedding and sectioning (3–5 μm). Sections were stained with hematoxylin and eosin (HE), a PAS staining kit or Masson’s Goldner Trichrome (all Carl Roth Karlsruhe, Germany) according to the manufacture’s protocols for histological evaluation. Prior immunofluorescent staining, samples were prepared by using a descending alcohol series and incubation with target retrieval solution (DAKO). Afterward, slides were blocked with a 2% normal goat serum/ PBS blocking solution to reduce unspecific interactions, and primary antibodies were incubated overnight at 4 °C. Antigens were detected by fluorescently labeled secondary antibodies. Nuclei were counterstained with DAPI.

### Radiation treatments

Irradiations of LuOrgs were performed at room temperature using an Isovolt-320-X-ray machine (Seifert-Pantak) at 320 kV, 10 mA, and a 1.65-mm aluminum filter at a distance of 50 cm with a dose rate about 3 Gy/min and energy of the tube at 90 kV (∼ 45 keV X-rays).

### Flow cytometry

Flow cytometric measurements were performed as previously described [12–14]. Single cell suspensions were generated by re-suspending LuOrgs in TrypLE (Thermo Fisher Scientific; #12604013) containing 100u/ml DNase I followed by a 15-min incubation at 37 °C. Digestion was stopped by adding PBS containing 2–5% fetal calf serum (FCS), 2mM EDTA and DNase I. The cellular solution was passed through a 70 μm cell strainer into a fresh conical tube prior downstream analysis. For each FACS staining reaction, 1 × 10^5^ cells were incubated with fluorochrome-coupled antibody (antigen-specific or isotype control) in 100µL FACS buffer (5% FCS in PBS) for 20 min at 4 °C. Cells were washed twice and subsequently re-suspended in 200 µl FACS buffer and analyzed on a CytoFLEX Platform (Beckman Coulter) using the CytExpert Software (Beckman Coulter). Cell cycle phases were analyzed at 96 h post-RT using respectively harvested cells (by trypsinization) in combination with Nicoletti staining solution [50 µg/mL 7-Aminoactinomycin D (7-AAD), 0.1% sodium citrate (w/v), and 0.05% Triton X-100 (v/v) in PBS] (30-min incubation at room temperature prior analyses). Senescence formation was analyzed four days post RT following bafilomycin A1 (100 nM, Biozol, Eching, Germany) and C12FDG (5-dodecanoylaminofluorescein di-β-D-galactopyranoside; 33 µM; Thermo Fischer Scientific Waltham, MA, USA) incubations.

### Bulk RNA sequencing analysis

Total RNA was isolated from cultures following sham (Ctrl, 0 Gy) or 10-Gy irradiations at 96 h post-RT and processed as previously described [13, 15]. In brief, RNA concentration and quality were measured with Qubit (Invitrogen, Waltham, MA, USA) and Agilent Bioanalyzer DNA HS (Agilent, Santa Clara, CA USA). Real-time RT-PCR quantifications following cDNA synthesis using QuantiTect Reverse Transcription (Qiagen, Hilden, Germany) were performed according to the manufacturer’s instructions and were carried out using specific deoxy-oligonucleotide primers. Library preparation was performed with Lexogens QuantSeq 3′ mRNA-Seq Library Prep Kit FWD and sequenced on a NextSeq500 (Illumina, San Diego, CA, USA). Sequences were trimmed with TrimGalore with standard settings and aligned with hisat2 to hg38 with standard settings. Statistical analysis was performed with R using the R-packages DESeq2 for main DEG calculations, ComplexHeatmap for heatmaps, umap for dimensionality reduction, fgsea for gene set enrichment analysis, and EnhancedVolcano) for volcano plots as previously described [13, 15].

### Single cell RNA sequencing analysis

For scRNAseq, dissociated LuOrgs were labelled using the BD^®^ Hu Single-Cell Multiplexing Kit (BD, Bioscience; #633781) according to the manufacturer’s instructions. Samples were afterwards combined (Sample 1 CTRL + Sample 1 XRT and Sample 2 CTRL + Sample 3 CTRL + Sample 2 XRT + Sample 3 XRT) and loaded on two lanes on a BD Rhapsody HT Xpress System aiming for 40.000 cells. BD Rhapsody library prep was performed according to the manufacturer’s instructions and sequenced on a NextSeq 2000 P3 100 cycles. BDs cwl-runner was used to align and pre-analyse the data. In total 14,937 cells (8696 CTRL and 6241 XRT) in both lanes could be identified. All further analyses were conducted with R packages (Seurat v.4, Enhanced Volcano, scPubr, fgsea, nVennR).

### Statistical analysis

If not otherwise indicated (n = biological replicates), data were obtained from at least three independent experiments. Data were presented as median values and Interquartile range (IQR). Data analyses were performed by two sided Mann-Whitney U tests in case of unpaired data or by Wilcoxon signed-rank test using R (R Core Team). Statistical significance was set at the level of *p* ≤ 0.05.

## Results

**Fig. 1:**
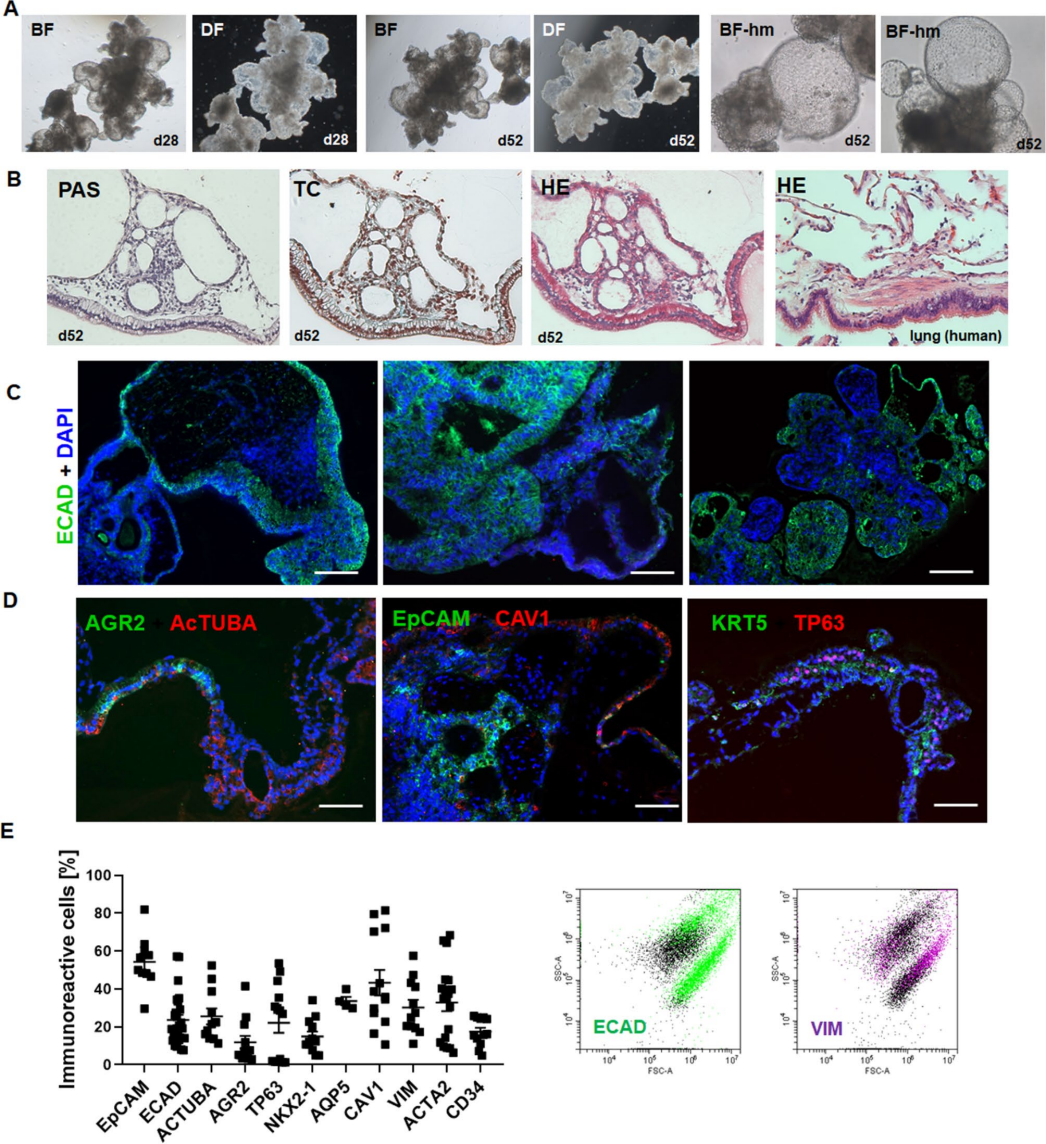
Morphological characterization of free-floating iPSC-derived human LuOrgs confirmed the efficient generation of desired lung structures and cell types. (**A**) Bright-field (BF) and dark-field (DF) images of LuOrgs generated matrix-free from human iPSCs in ultra-low attachment plates at indicated time points using the 5x and 20x objective of an inverted microscope. (**B**) Histology staining of paraffin-embedded sections of the lung-like regions of LuOrgs using Periodic Acid Schiff (PAS), trichrome (TC), and hematoxylin and eosin (HE) staining. Normal human lung tissue was included as positive control. Magnification: 10x. (**C**) Immunofluorescence staining of paraffin-embedded sections of LuOrgs representing different the lung-like regions for the lung epithelial markers ECAD (green). Nuclei were counterstained using DAPI (blue). Scale bar: 200 μm (**D**) Representative immunofluorescent images of indicated lung cell type-marker proteins. Scale bar: 100 μm. (**E**) Flow cytometry quantification of epithelial and mesodermal lung cell types of single cell dissociated LuOrgs (d45-52). Data are shown as means ± SEM of indicated biological replicates. Representative FACS plots of an epithelial and mesodermal marker is shown. See also Figure S1

We established a simple protocol for the generation and basement matrix-free cultivation of iPSC-derived lung organoids (LuOrg) in ultra-low attachment plates through embryoid bodies and subsequent branching lung organoid (BLOB) media treatment (Fig. 1, Supplemental Figure S1). During the differentiation process, lung organoids gradually form from the generated spherical embryoid bodies, with lung budding and branching becoming prominent around 14 days post plating (Supplemental Figure S1). After approximately 28–52 days of cultivation, lung organoids can be obtained that consist of a polarized, pseudo-stratified airway epithelium. Morphological analyses by phase contrast microscopy (Fig. 1A, Supplemental Figure S1), immunohistochemistry (Fig. 1B, Supplemental Figure S1) and immunofluorescence (Fig. 1C, D Supplemental Figure S1) further confirmed the efficient generation of airway and alveolar structures representing the desired destination tissue: the human lung. Similar to protocols that use four stages of differentiation, namely definitive endoderm, anterior foregut endoderm, which in turn were Matrigel embedded and then subjected for lung bud organoid differentiation finally resulting in and branching lung organoids (not shown), the free-floating LuOrg generated here formed patterned branch-like structures with interior regions that express proximal airway markers, and distal bud tip regions. Flow cytometry analysis was further used to confirm presence of different epithelial and mesenchymal lung cell type (Fig. 1E, Supplemental Figure S1) and to quantify various cell types, including airway (basal, ciliated, and goblet cells), alveolar (alveolar epithelia cells, AECI/II) and fibroblasts after single-cell dissociation.

**Fig. 2:**
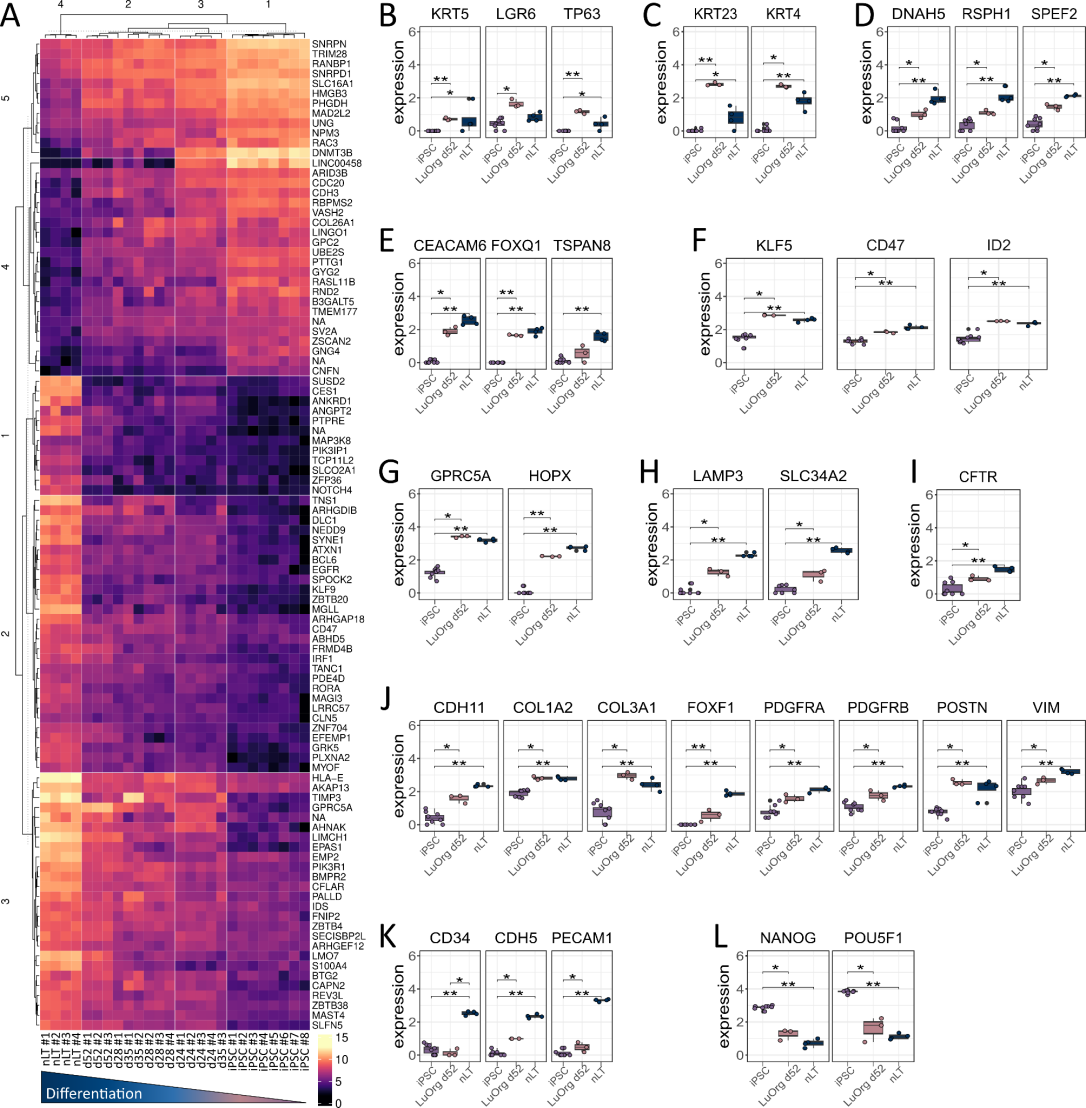
Molecular characterization: RNA sequencing analysis verified directed differentiation of human iPSCs into lungs cells. (**A**) Heatmap of top normalized differentially (padj < 0.001, abs(log2FC) > 0.5) expressed genes of indicated 26 samples in different differentiation stages (d0/iPSCs (8 samples), d28 (4 samples), d35 (3 samples), d52 (3 samples)) compared to normal lung tissue (nLT (4 samples)). Biological replicates as indicated. Heatmap is row-normalized to show differences between the samples. (**B**)-(**L**) Normalized expression of iPSCs, generated lung organoids at d52 (LuOrgs d52) and nLT signature genes for (**B**) basal, (**C**) suprabasal, (**D**) ciliated, (**E**) goblet, (**F**) alveolar, (**G**) AECI, (**H**) AECII, (**I**) ionocyte, (**J**) fibroblastic, (**K**) endothelial and (**L**) pluripotent cells. Mann-Whitney 𝑈 test. * = 0.05, ** = 0.01, *** = 0.001. See also Figure S2

To confirm directed differentiation of human iPSC clones into LuOrg, we performed serial quantitative gene expression analyses at day 0 (iPSCs), 24, 28, 35 and 52 compared to normal lung tissue (nLT) to determine the degree of lung development (Fig. 2, Supplemental Figure S2). A hierarchical clustering was built based on expression levels of gene identifying signatures (with log-fold change > 0.5 and adjusted *p*-value < 0.001) that validated the proximity of the generated lung structures (d52) to the target tissue (Fig. 2A). Gene set enrichment analysis (GSEA) according to the TRAVAGLINI [16] and HE_LIM_SUN [17] lung cell gene sets from the molecular signatures database MSigDBv6.1 (Broad Institute) confirmed successful generation of the most relevant lung cell types while immune subsets were lacking (Supplemental Figure S3). Beside the morphological examinations, the molecular analyses showed no great difference between d28-d52 and d28, i.e. differentiation has already been sufficiently completed, which, in addition to its practicality, also indicates a very favorable (short) time window to broaden the methodology in general. However, we chose the latter time point for further analyses to be consistent with the already known time points and thus ensure sufficient differentiation into the desired cell types. Comparative analyses of relative RNA levels further highlight significantly upregulated expression levels of typical airway cells including basal (KRT5, TP63, LGR6), parabasal (KRT4, KRT23), ciliated (DNAH5, RSPH1, SPEF2) and secretory/goblet (FOXQ1, CEACAM6, TSPAN8), ionocyte (CFTR) cell markers, as well as significantly upregulated expression levels of the alveolar lineage maker KLF5, CD47 and ID2 together with the AECI (HOPX, GPRC5A), and the AECII (LAMP3, SLC34A2) marker genes (Fig. 2B-I, Supplemental Figure S2). Significant upregulated expression levels of mesodermal genes include CDH11, COL1A2/1A3, FOXF1, PDGFRA/B, VIM and POSTN (Fig. 1J) while a lower, but also significant upregulation of endothelial genes was detected (Fig. 2K). As expected, significant declines in NANOG and POU5F1/OCT4 expression were detected (Fig. 2L).

**Fig. 3:**
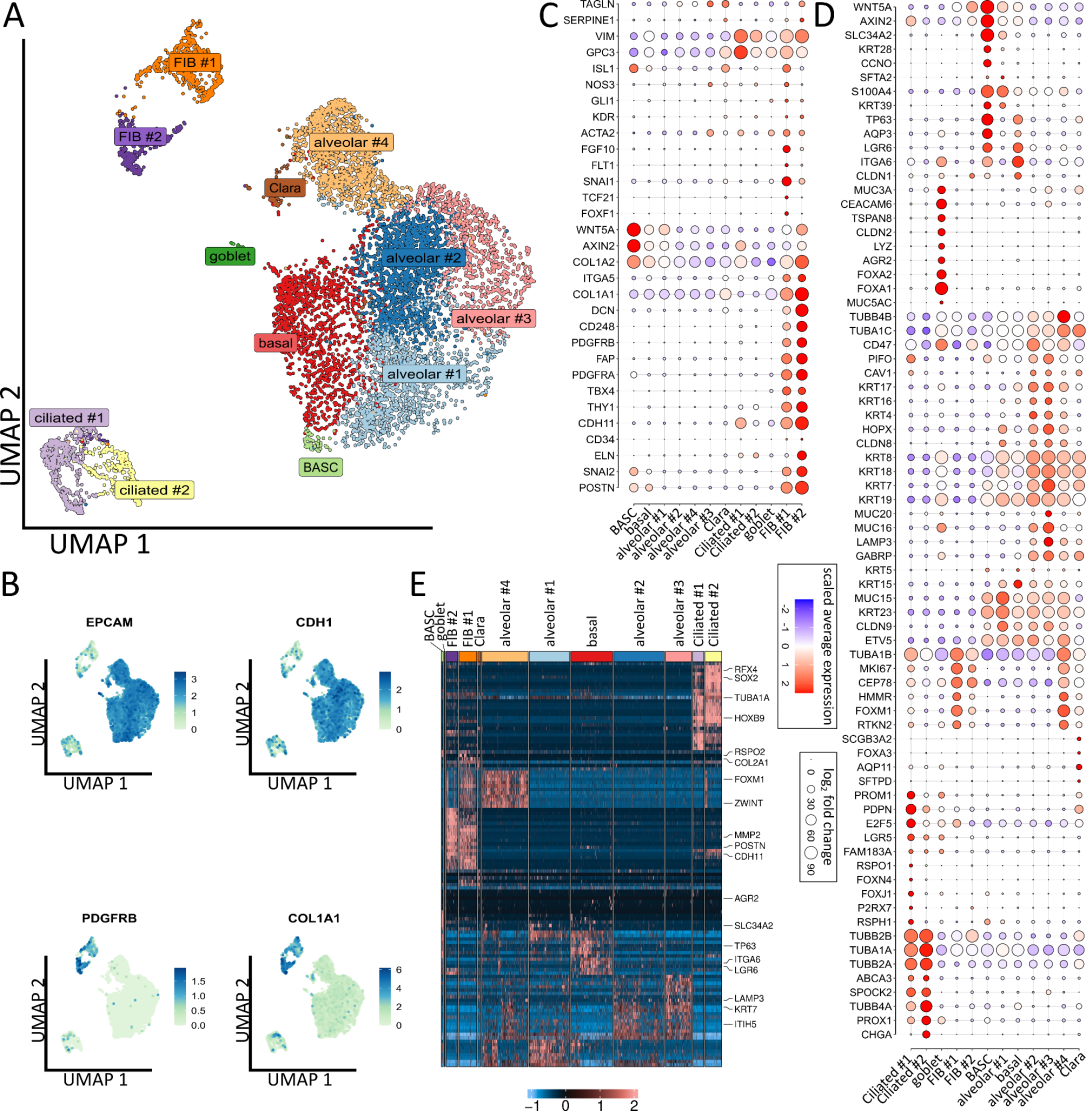
Single cell analysis revealed the composition of lung organoids. (**A**) Uniform Manifold Approximation and Projection for Dimension Reduction (UMAP) of all single cells colored by identified clusters. *N* = 3 biological replicates. Cell-type labels for each cluster are based on expression of canonical cell-type markers displayed in the dot plot in C, D or Fig. S4B (**B**) Expression map of representative epithelial (EPCAM, CDH1) and mesenchymal (PDGFRB, COL1A1) marker genes in all clusters. (**C**) Dot plot of indicated mesenchymal marker genes. Size of the dots indicate the percentage of cells in which this gene was found, the color indicates the normalized value of the expression. (**D**) Dot plot of epithelial marker genes. (**E**) Row-normalized heatmap of top ten differentially expressed genes per cluster. Important genes are indicated by name. See also Fig. S4

Single cell RNA-seq of LuOrgs (d52) generated from two different iPSC cell lines was performed to finally proof presence of the different epithelial and mesodermal lung cell subsets and characterize respective phenotypes in more detail (Fig. 3, Supplemental Figure S4). Clustering of the single cells revealed the presence of 12 different main cellular subsets (Fig. 3A), including epithelial and mesodermal lung cells as highlighted by the distribution of epithelial EpCAM/CD326, ECAD/CDH1, PDGFRB and COL1A1 expressions (Fig. 3B). Among the different subsets, basal cells expressing ITGA6, TP63, and LGR6 as well as bronchioalveolar stem cells (BASCs) were identified (Fig. 3C-D). BASCs usually locate at bronchioalveolar duct junctions and are characterized by co-expression of bronchiolar (CCNO) and alveolar (SLC34A2, AXIN2) epithelial genes together with TP63, LGR6, AQP3 and WNT5A, the latter one being known to regulate distal lung morphogenesis. Two TUBA1A and TUBB2B expressing ciliated (#1, #2) cell clusters were identified, with cluster ciliated #1 (RFX4, FOXJ1, RSPH1) comprising (differentiating) luminal epithelial cells undergoing ciliogenesis and with cluster ciliated #2 comprising more mature ciliated cells (TUBB4A, PROX1, KIF5C). Secretory cells and particular goblet cells were identified by the expression of LYZ, ARG2, MUC3A, TSPAN8 and CEACAM6 together with FOXA1 and FOXA2, close to designated Clara (club) cells expressing SCGB3A2, SFTPD and FOXA3. Four alveolar and two fibroblastic clusters were further identified (Fig. 3A, C-D, Supplemental Figure S4). Cluster alveolar #1 consist of AECI/II lineage-prone cells (AECI/II-differentiating BASC cells) expressing HOPX, ETV5, SLC34A2, KRT23 and MUC15. More advanced differentiated alveolar cells were designated based on the additional expression of GABRP, LAMP3 and CAV1 (alveolar #2), beside AECII (alveolar #3) expressing increased LAMP3 and GABRP levels, together with high molecular weight glycoproteins (MUC16, MUC20), and GDF15 while HOPX levels were reduced. The alveolar #4 cluster was characterized by FOXM1, RTKN2, ZWINT expression and was designated as AECI cluster in direct vicinity to alveolar fibroblasts (FIB #1) expressing FOXM1 together with FGF10, SNAI1, FOXF1 (together with classical mesodermal genes). Adventitial fibroblasts (FIB #2) showed increased expression levels of DCN, ELN, FAP, POSTN and CD248/ TEM1 and thus more reactive ECM-remodeling related genes. Based on the expression of MKI67 an HMMR, proliferation was prominent in the FIB#1 (alveolar fibroblasts) and the alveolar #4 (AECI) clusters. The absence or pluripotency-related (NANOG, POU5F1) and hematopoietic lineage (PTPRC/CD45) expressions became obvious in respective UMAP plots, while only single endothelial cells as indicated as VECAD/CDH5, VEGFR1/FLT1 or eNOS expressing cells (interspersed in alveolar #1, #4 cluster) were detected (Supplemental Figure S4).

**Fig. 4:**
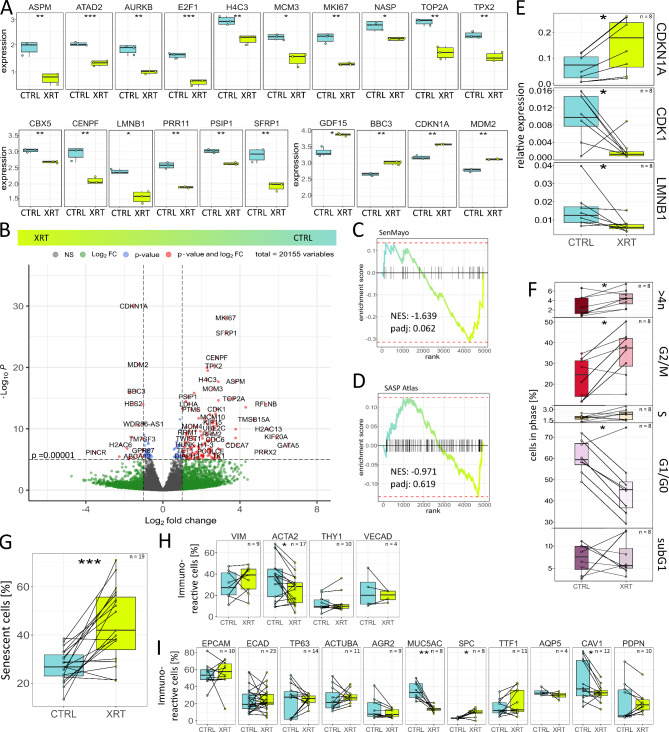
Modeling lung injury: senescence as prominent radiation response within LuOrgs. (**A**) Expression pattern changes in lung organoids after irradiation (bulk RNAseq). Normalized expression of control (CTRL) and irradiated (XRT) lung organoids at d52 (96 h post RT with 10 Gy) of the 20 most affected genes following RT are depicted (*n* = 3 biological replicates per group). (**B**) Volcano plot of all genes. Labels are added to genes which have an absolute log2 fold-change higher than 1.5 and a *p*-value lower than 0.00001 (Wald test). (**C**) Enrichment plots of a geneset enrichment analysis of the SenMayo gene set identifying senescent cells across tissues [18]. *P*-values are calculated by adaptive multi-level split Monte-Carlo scheme (fgsea R-package). (**D**) Enrichment plots of a gene set enrichment analysis of the SASP Atlas gene set comprising soluble proteins and exosomal cargo SASP factors exclusive to ionizing radiation [19]. *P*-values are calculated by adaptive multi-level split Monte-Carlo scheme (fgsea R-package). (**E**) Indicated transcript levels were quantified using Real-Time RT-PCR and are shown as relative expression to beta-actin. (**F**) Cell cycle phases and apoptotic cells (subG1) were analyzed by flow cytometry in LuOrgs following single cell dissociation. See methods for details. (**G**) RT-induced senescence formation was analyzed by C12FDG staining prior to flow cytometry analyses. Flow cytometry quantification of mesodermal (**H**) and epithelial (**I**) lung cell types of single cell dissociated LuOrgs. Biological replicates as indicated. Mann-Whitney 𝑈 test. * = 0.05, ** = 0.01, *** = 0.001. See also Figure S5 and S6

We wanted to test the quality and robustness of our model as a potential in vitro platform for lung diseases, namely radiation-induced lung injury. Therefore, the radiation response of cultured LuOrg was analyzed 96 h after RT treatment with 10 Gy (Fig. 4). First bulk RNAseq analyses were performed to get an idea about the affected signaling pathways following RT. Among the most affected pathways following RT, cell cycle-related processes were affected as well as genes involved in the DNA damage response and thus cellular fate (Supplemental Figure S5). The overall proliferation-associated genes (e.g., AURKB, E2F1, H4C3, MCM3, MKI67 and TOP2A) were the most down-regulated genes and BBC3, CDKN1A, GDF15, MDM2 were the most up-regulated genes (Fig. 4A). The combination of down-regulation of the proliferation marker MKI67, the nuclear lamina intermediate filament LMNB1 and up-regulation of the potent cyclin-dependent kinase inhibitor CDKN1A, MDM2 and GDF15, a core senescence associated secretory phenotype (SASP) factor, might indicate cellular senescence. We therefore used the ‘SenMayo’ gene set for the identification of senescent cells and prediction of senescence-associated pathways across tissues [18] to investigate potential RT-induced senescence in our LuOrg (Fig. 4B, C). GSEA revealed a strong (but not significant) trend for senescence while no clear enrichment of secretory SASP factors exclusive to ionizing radiation [19] could be detected (Fig. 4D). We confirmed RT-induced senescence using quantitative Real Time RT-PCR by upregulation of the senescence marker CDKN1A and LMNB1 while the mitotic cell cycle regulator CDK1 was down-regulated (Fig. 4E) and by Westernblot and immunofluorescent analysis of respective marker proteins (Supplemental Figure S6). A G2/M cell cycle arrest but no significant cell death/apoptosis induction as indicated by increasing subG1 fractions could not be observed (Fig. 4F). Flow cytometry in combination with the fluorescent dye C12FDG, which can be hydrolyzed by β galactosidase enriched in lysosomes upon senescence yielding in increased green fluorescence, showed a significant increase in senescent cells (Fig. 4G). Flow cytometry analyses of cellular alterations within LuOrgs following RT (Fig. 4H, I) further revealed no obvious alterations concerning the cell numbers of EpCAM- and ECAD-positive epithelial cells as well as for TP63-positive basal and AcTUBA-positive ciliated cells. Goblet (AGR2, MUC5AC) and AECI (CAV1, PDPN) cells were reduced in numbers and SPC- and NKX2-1/TTF1-positive AECII cell numbers were increased. Within the mesodermal cells, reduced numbers of ACTA2-positive cells could be detected, while the numbers of VIM-immunoreactive cells remained unaffected.

**Fig. 5:**
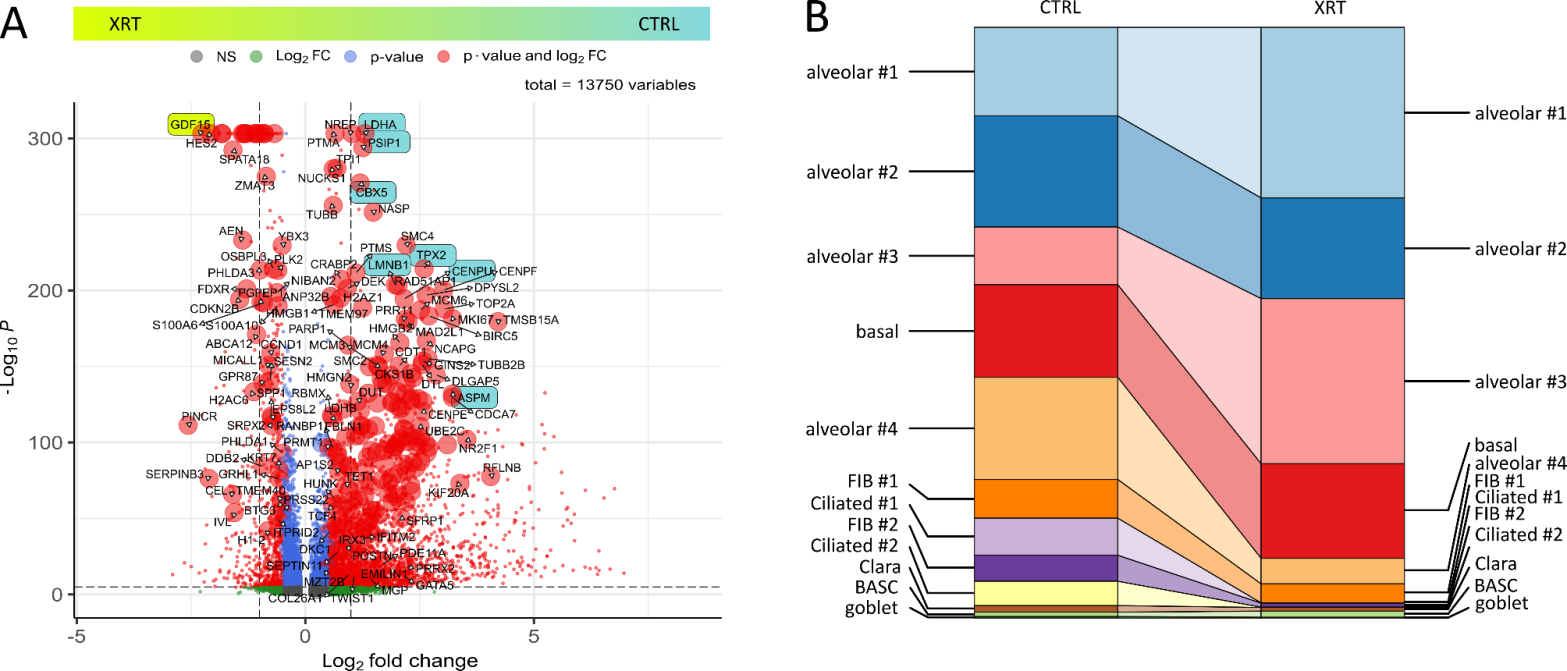
Single cell analysis reveal a differential RT response in LuOrgs with the airway epithelium being most sensitive. (**A**) Volcano plot of all differentially expressed genes (cluster-independent) obtained from scRNAseq data sets (*n* = 3 biological replicates, Mann-Whitney 𝑈 test). Labels are added to genes which have an absolute log2 fold-change higher than 1.5 and a *p*-value lower than 0.00001. Top differentially expressed genes from the bulk RNAseq depicted in Fig. 2A are additionally colored in green or blue and all identified genes depicted in Fig. 4B are highlighted by increased dot size. (**B**) Changes of cluster sizes due to irradiation

To further gain insight into the cell-type specific molecular alterations following RT and particular with respect to single cellular fates, single cell RNA-seqs of irradiated LuOrgs were analyzed (Fig. 5). Affected genes following RT as estimated from the bulk RNA-seq data could be confirmed, but also additional genes were identified (Fig. 5A). Changes of cluster sizes due to RT revealed increases in portion of cells of the alveolar #1 (AECI/II lineage-prone cells) and alveolar #3 (AECII) clusters, while the portion of the alveolar #2 (more advanced differentiated alveolar cells) cluster -similar to the basal cell and BASC clusters- remained unchanged, and the alveolar #4 (AECI) cluster being decreased (Fig. 5B). A slight decrease was also estimated for the alveolar FIB #1 cells, while the portion of adventitial FIB #2 cells decreased more. Similarly, decreases in the ciliated, the Clara and goblet cell clusters could be observed with the latter ones showing at all very low abundances (Fig. 5B).

**Fig. 6:**
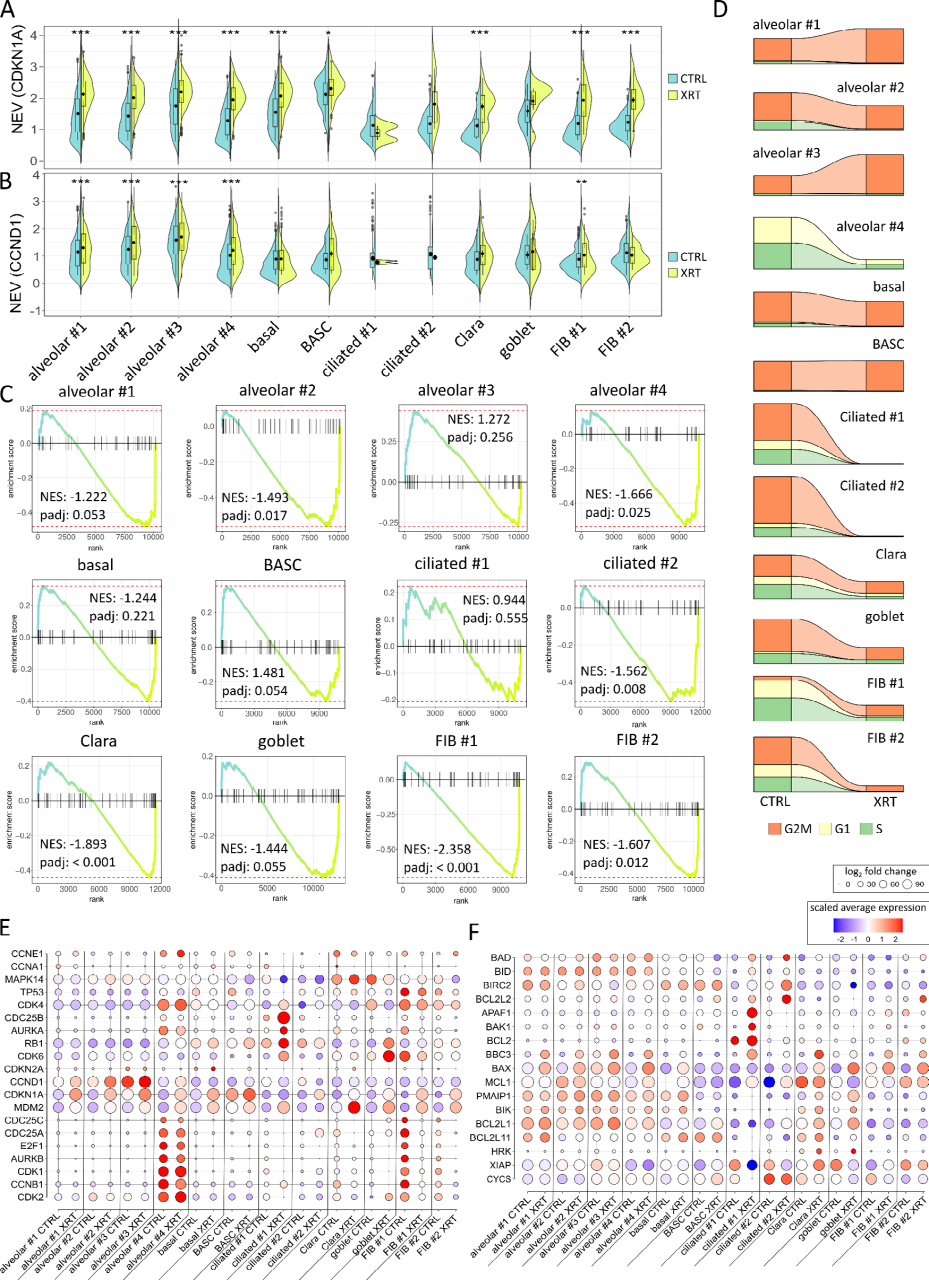
Analysis of the cell-type dependent senescence induction revealed a senescent phenotype in fibroblasts, Clara and goblet cells following RT. Normalized expression values of the senescence marker as violin plots of (**A**) CDKN1A and (**B**) CCND1 of each cluster before (CTRL) and after irradiation (XRT) for each cells expressing the marker. (**C**) Enrichment plots of a gene set enrichment analysis of the SenMayo gene set for each cluster. *P*-values are calculated by adaptive multi-level split Monte-Carlo scheme (fgsea R-package). (**D**) Cell cycle classification per cluster before and after RT. (**E**) Expression of cell cycle genes in each cluster before and after irradiation. (**F**) Expression of apoptosis-related genes in each cluster. Size of the dots indicate the percentage of cells in which this gene was found, the color indicates the normalized value of the expression. See also Figure S7

Concerning the cellular fate in response to RT, and particularly with respect to RT-induced senescence, the molecular alterations were investigated (Fig. 6, Supplemental Figure S7). While all clusters showed an increase in CDKN1A levels following RT, significant increases in CCND1 levels could only be detected in all alveolar and the FIB #1 clusters (Fig. 6A, B). Significant enrichment of genes of the ‘SenMayo’ senescence signature could been estimated for the alveolar #1, #2, #3, the ciliated #2, the Clara and goblet, and both FIB clusters (Fig. 6C). Thus, RT-induced senescence could potentially be claimed for these clusters, keeping in mind that both ciliated as well as the alveolar #4 (AECI), both FIB, and the secretory (Clara, goblet) cells decrease following RT. In order to eventually unravel the mechanism behind the potential senescence induction, cell cycle classification per cluster before and after irradiation was performed together with gene expression analysis of cell cycle and senescence-associated genes as well as of apoptosis-related genes as revealed from the single cell RNA-seq data sets (Fig. 6D-F). According to the reported increase of the alveolar #1 and alveolar #3 clusters, increases in G2/M phases could be detected, while G2/M phases remained stable in the alveolar #2, the basal and the BASC clusters with vanishing (of the very low) G1 phases in the alveolar #2 and basal cell clusters (Fig. 1D). Decreases of the alveolar #4 and FIB portions came along with decreases in all cell cycle phases. Within the FIB #1 cluster an increased G2/M portion became prominent following RT and being indicative for a G2/M arrest. As ciliated cells diminished in response to RT, no phases could be estimated here. Clara and goblet cells showed - according to the decreased cluster portion - also reduced phases following RT. Beside the induced expression of CDKN1A and CCND1 in the alveolar clusters, increased levels of anti-apoptotic BCL2L1/BCL-xL could be observed for the alveolar clusters, which was accompanied by increased pro-apoptotic BAX and BBC3/PUMA levels (Fig. 6E, F). The latter ones also were found upregulated in alveolar #4 and FIB#1 cluster, together with a reduction of cell cycle progression genes in the FIB #1 cells. Thus, RT-induced cell death via apoptosis might be the major prominent cell fate within these clusters. Similarly, ciliated #1 cells showed increased pro-apoptotic BAX together with increased APAF1 levels that could account for the reduced portion of cells within this cluster post RT. Within the Clara cells, elevated levels of MDM2 and MAPK14 could be observed, while goblet cells showed increased CDK6 levels generally acting the interface of TP53 and RB by driving cell-cycle progression and antagonizing stress responses. No obvious alterations concerning cell cycle and apoptosis-related genes could be observed for basal cells, BASC, alveolar #3, FIB#2 and ciliated #2 cluster cells, although the latter ones severely deceased in response to RT. Thus, based on RT-induced CDKN1A and CCND1 levels, together with the significantly enriched genes of the ‘SenMayo’ signature and the cell cycle-related changes, RT-induced senescence could be stated for the alveolar cells particular for the alveolar #2 subset, namely AECI/II lineage cells. AECI/II lineage-prone cells (alveolar #1 cluster) showed also these senescence characteristics but increase in numbers, a fact that might be indicative for regenerative processes, together with the increase of the AECII (alveolar #3) cluster that lacks a clear senescent phenotype. Both ciliated cell clusters showed the strongest decrease following RT, together with AECI (alveolar #4) cells, which might reflect the highest radiation sensitivity of these cells within LuOrgs. In contrast basal cells and BASC were not severely affected by RT, nor concerning a senescent phenotype or their numbers. The reduced portions of both FIB, the Clara and the goblet cell clusters showed a clear senescent phenotype following RT.

**Fig. 7:**
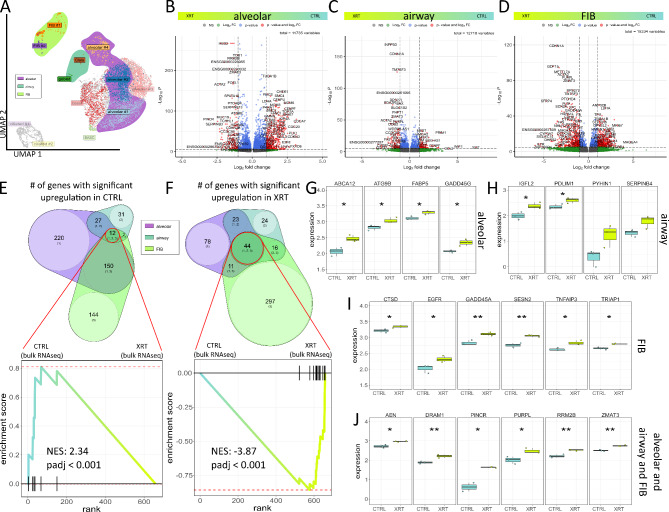
Potential RT-induced senescence signatures in lung organoid meta clusters. (**A**) Uniform Manifold Approximation and Projection for Dimension Reduction (UMAP) of all XRT single cells colored by identified clusters. *N* = 3 biological replicates. Meta cluster are shown by respective color. Volcano plot of all genes in (**B**) alveolar (comprising the alveolar clusters #1, #2, and #4) (**C**) airway (Clara and goblet) and (**D**) FIB (#1 and #2) meta cluster. Labels are added to genes which have an absolute log2 fold-change higher than 1.5 and a *p*-value lower than 0.00001 (Mann-Whitney 𝑈 test). Venn diagrams of significantly up regulated genes in CTRL (**E**) and RT-treated (**F**) LuOrgs meta clusters and enrichment plot of the bulk RNAseq data sets before and after RT. *P*-values are calculated by adaptive multi-level split Monte-Carlo scheme (fgsea R-package). Normalized expression of 3 biological replicates of control (CTRL) and irradiated (XRT) LuOrgs at d52 in respective bulk RNAseq for genes differentially expressed in single cell alveolar (**F**), airway (**G**) and FIB (**H**) meta clusters, and intersection of all three (**I**). Mann-Whitney 𝑈 test. * = 0.05, ** = 0.01, *** = 0.001. See also Figure S8, S9 and Table S1

Based on these results we finally investigated the molecular changes concerning the senescent alveolar, airway (Clara and goblet cells) and FIB meta clusters in order to unravel and highlight RT induced changes that could be used to understand the alterations of RT-induced lung injury (Fig. 7, Supplemental Figure S8-9). Volcano plot analysis visualized differentially expressed genes in meta clusters (Fig. 7A-D). Using the log-fold change > 1.5 and the adjusted *p*-value of < 0.00001, 298 transcripts (logFC > 1.5, *p*-value < 1*10^− 5^) were found to be differentially expressed in both the alveolar cluster, with 78 genes found to be upregulated in response to RT compared to 220 upregulated genes in Ctrl (non-irradiated) cells (Fig. 7E, F). Among those genes, the CD47-THBS1 signaling axis turned out to be critically involved (Supplemental Figure S8). Differentially expressed genes in the senescent airway clusters comprised 24 genes found to be upregulated following RT compared to 31 upregulated genes in Ctrl and 207 upregulated genes in the irradiated fibroblast subset compared to 144 upregulated genes in respective control. Expressions, particularly of upregulated genes following RT in alveolar, airway, and FIB meta clusters as well as 44 upregulated genes in the intersection of all three was further confirmed in the bulk RNAseq data sets (Fig. 7G-J, Supplemental Figure S9, Table S1). Beside 8 alveolar, 5 airway, and 28 FIB candidate genes, a intersecting gene signature consisting of 15 genes was found to be induced in LuOrgs after irradiation, which could be used as additional as well as lung cell type-specific senescence gene sets.

## Discussion

Platforms based on patient-derived organoids, but also on organoids from self-organizing, expanding iPSC 3D cultures, offer an unprecedented opportunity to specify relevant strategies for radioprotection of normal tissue while excluding tumor-promoting side effects. Responses and results of modulating therapeutic substances, validation of clinical trials, and ultimately patient care can thus be optimized. The practicality of the studies should be emphasized through appropriate studies on commercially available cell lines if not every laboratory has human tissue samples and/or iPSC technology available. Here, we used a very simple and easy to use approach to generate successfully and efficiently lung organoids from iPSCs without relying on the use of Matrigel, one of the original and most widely referenced but animal-based 3D matrices.

Based on the donor cell type as source of their starting material, three different types of lung organoids are currently used for modeling lung physiology and disease. Fetal and adult lung stem cells as well as iPSCs-derived organoids, which were generated and maintained for long-term culture in the relatively undefined mouse matrix Matrigel in combination with optimized media formulation [20]. For the latter ones, branching lung organoids were obtained from human induced iPSCs through four different differentiation stages finally generating lung organoids in up to 85 days [8]. Here we used the branching lung organoid medium described by Miller et al. [8] to foster embryoid bodies differentiation into the presented epithelial and mesodermal lung cell types, which in turn were spontaneously generated from iPSCs in the suspension system using ultra-low attachment plates. Generally, the multi-germ-layer involvement for LuOrg development is of particular importance, as mesenchymal-epithelial interactions are indispensable for normal lung development, homeostasis, and regeneration, and thus for LuOrg generation and maintenance especially for alveolar structures, although the simultaneous differentiation of lung epithelial and mesenchymal cells remains challenging [21, 22]. The use of EB-mediated differentiation as performed in our studies has circumvented this challenge. Resulting LuOrgs developed proximal- and distal-like branching airway epithelial structures within 28-52d. We chose the latter time point for our analyses to be consistent with the already known time points and thus ensure sufficient differentiation into the desired cell types. However, the morphological and molecular analyses showed that there is no great difference between both periods, i.e. differentiation has already been sufficiently completed.

### Modeling lung injury

Respiratory diseases include a broad spectrum ranging from acute lung injury, such as pneumonia or lower respiratory tract infection, to chronic diseases, such as pulmonary fibrosis and chronic obstructive pulmonary disease, and are among the most common causes of illness, disability, and death worldwide [23, 24]. The molecular and cellular dynamics of the lung -a highly specialized organ- as well as respective alterations observed within respiratory diseases must be understood in more detail, including the identification of novel signaling pathways, in order to prevent the development of injury and/or unravel additional and new druggable targets for treatment. Lungs are further among the most sensitive organs to ionizing radiation and this high intrinsic sensitivity is dose-limiting for thoracic radiotherapy (RT) [25]. The progressive process of RT-induced lung injury (RILI) includes acute inflammation and repair, which can develop into chronic inflammation and pulmonary fibrosis [26]. Classical preclinical models for investigating normal tissue damage are mouse models of radiation-induced pneumopathy. But this and other animal models only inadequately reflect the clinically relevant dose sensitivities and the relevant characteristics of human RILI pathophysiology, which ultimately limits their value [27]. For example, the RILI mouse models usually show only mild pneumonitis and minimal lung fibrosis, which usually results from a single-stage irradiation rather than a fractionated irradiation regime. In addition to long breeding periods, an extremely time-consuming radiation experiment due to very long treatment and survival times and thus high costs, serious ethical concerns are increasing significantly today [28–30]. Corresponding clinical studies are practically impossible for ethical reasons, particularly due to (medical) radiation protection measures. Therefore, alternative patient-oriented preclinical models are urgently needed to better understand the molecular basis in relation to the human system in order to then evaluate the effectiveness of new therapeutic approaches [28, 31].

As a potential in vitro platform for lung diseases, we investigated quality and robustness of our LuOrgs to model radiation-induced lung injury. We followed particular the different cell fates following RT, including senescence and the associated hypersecretory phenotype of senescent cells, which are central aspects in the pathology of RILI [32, 33]. We found similar observations as already known from animal models and from the (limited) human investigations mainly derived from retrospective immunohistological investigations. In RILI, generally five different phases ranging from acute pneumonitis to chronic pulmonary fibrosis can be distinguished [34]. Accordingly, we observed decreases in AECI cells (alveolar #4 cluster), while the portion of AECI/II lineage-prone cells and AECII (alveolar clusters #1 and #3) increased, consistent with the early phases [34–36]. A severe decrease in the ciliated cell clusters could be observed following RT, together with the reductions in Clara and goblet cell clusters, while the remaining Clara and goblet cells showed a senescent phenotype, which are indications for the late phase [34, 37]. Murine experiments in our own group showed the induction of senescence through RT-induced damage to airway (bronchial) epithelial cells [38, 39]. Our model might not reflect the exudative [34, 40], intermediate [34, 41] and resolution phase [26, 34]. Only single endothelial cells (interspersed in the alveolar #1 cluster) could be detected within LuOrgs and we detected only a slight decrease for alveolar FIB #1 cells, while the portion of adventitial FIB #2 cells decreased more, both populations exhibiting a senescent phenotype following RT. Of note, no reductions indicating no RT induced cell death (apoptosis) as well as no senescence characteristics could be observed in the BASC and basal cell population, indicating potentially a less radiosensitive phenotype, or a different RT-induced cell fate, e.g. differentiation into alveolar cells.

### The overall RT response of LuOrgs

Epithelial cells show critical secretory and regenerative roles to maintain lung homeostasis. In combination with the loss of airway epithelial cells following RT, the altered secretory phenotype of remaining, senescent cells accounts for the development of a pro-inflammatory and -fibrotic phenotype. Similarly, the fibroblasts subsets were affected. RT-stressed alveolar cells showed less reduction concerning the cell numbers, which might already highlight their role concerning lung regeneration. The here presented gene signature of the intersection of alveolar, airway, and FIB meta clusters containing candidate genes being upregulated following RT could be used in the future for cell-type independent RT-damage.

We could identify several genes involved in the p53-MDM2 axis being differentially expressed after RT induction. Mouse double minute 2 (MDM2) molecules are activated by and an important negative regulator of p53 and showed an induction upon RT treatment. This highlights the possibility of restoring p53 function by inhibiting the interaction between p53 and MDM2 or by degrading MDM2, to increase p53 levels and thus altering cellular fates, e.g., cell cycle arrest and senescence, as well as survival and cell death following RT [42, 43]. Particularly concerning an altered lung function with reduced lung remodeling and regeneration, and thus an increased susceptibility to acute and chronic lung diseases following lung (premature) aging, suggests targeting the p53-MDM2 interaction as an useful option to limit normal lung damage while preventing tumor cell proliferation and survival [44, 45]. The importance of the p53-MDM2 axis for senescence is further highlighted from studies showing that MDM2 inhibitors could reduce the pro-inflammatory environment (e.g., reduced expression of the signature SASP factors IL-6 and IL-1α) created by senescent cells, thereby limiting disease progression [46]. In contrast, a central role of the MDM2-p53 axis was suggested for lung progenitor cells, where the p53-dependent DNA repair and cell survival system is more active in early progenitors and just differentiated cells compared to terminally differentiated cells, and therefore the p53 protein turnover by MDM2 is essential for the survival of respiratory progenitors [47]. Likewise, the p53-inducible gene ZMAT3 (also known as WIG-1 and encoding an RNA-binding protein) was induced following RT. ZMAT3 was reported to control splicing of mRNAs, including MDM2 [48]. Among the upregulated genes following RT, the apoptosis-enhancing nuclease (AEN), a known p53 target, was also identified. AEN was shown to be required for efficient DNA fragmentation in p53-dependent apoptosis [49]. AEN, together with three other p53-related genes (DDB2, TRIAP1, and TRAF4) combined serves as early biomarker for acute radiation injury [50]. Thus, targeting the p53-MDM2 axis might be a promising option for modulating the RT response of lungs. Finally, the growth differentiation factor 15 (GDF15) was found to be highly induced by RT. GDF15 turned out to be a general marker of cellular stress as the expression of GDF15 generally increases under pathological conditions, particularly in various chronic lung diseases [51]; and as a key SASP factor GDF15 was linked here to senescent cell states of lung epithelial cells [52].

### The airway epithelium-specific RT response

Ciliated cells, as revealed by substantial decreases following RT, might represent the most (radio) sensitive cells within lungs. Decreases of the Clara and goblet cells were even detected with a senescent phenotype of the remaining once. The altered secretome and in particular certain SASP factors identified here of senescent secretory cells after RT represent promising targets for anti-inflammatory and anti-fibrotic interventions. For example, expression levels of PYHIN1 were found to be increased after RT, a known regulator of pro-inflammatory cytokine induction in airway epithelial cells [53]. PYHIN family members, belongs to germline-encoded pattern recognition receptors, though which immune cells detect pathogens (innate immune response) [54]. Through binding to different cell surface receptors, particularly through interactions with toll-like receptor 4 and receptor for advanced glycation end products, these proteins activate mitogen-activated protein kinases (MAPK) responses and transcription factors, such as NF-*κ*B, resulting in the production of proinflammatory cytokines [55, 56]. Likewise, SERPINB4 (together with SERPINB3) also known as squamous cell carcinoma antigen-2 (SCCA2), can be linked to lung injury [57], namely inflammation where elevated levels of serpins could elicit a pro-inflammatory response and it and it has also been associated with the airway epithelium [58]. Furthermore, expression of fibrosis-related gene became upregulated. Insulin-like growth factors as well as their binding proteins are known key players in the development and progression of pulmonary fibrosis [59]. Particularly the insulin-like growth factor like family member 2 (IGFL2) was identified as differentially expressed gene in human idiopathic pulmonary fibrosis tissues correlating with lung function [60], and thus might represent a potential therapeutic target.

### The alveolar epithelium-specific RT response

As additional NF-*κ*B and thus senescence associated pro-inflammatory cytokines, genes from the S100 protein family triggering immune cell activation [55] were upregulated following RT with S100A9 in the airway and S100A8 in the alveolar subgroup. The S100 protein family is generally involved in pulmonary diseases and might even represent attractive therapeutic targets as S100 proteins mediate immune cell activation once released to the extracellular space [55]. In the alveolar group, increased levels of the autophagy-related protein 9B (ATG9B) known to contribute to the lung immune defense by regulating bacterial internalization via lung epithelial cells [61], to enhance protective autophagy and maintain stemness [62], and to modulate epithelial integrity by accelerating focal adhesion assembly [63], were further found. Likewise, an upregulation of GADD45G was observed. The DNA damage-inducible (GADD45) family generally regulates biological processes, such as cellular survival, cell cycle arrest and senescence as well as the inflammatory response following genotoxic stress [64], with GADD45G mediating G2/M cell cycle arrest [65] as reported here. Another important molecule concerning the alveolar RT response is CD47, also known as also known as integrin-associated protein. As an already known alveolar signature gene [66] playing a decisive role for effective derivation of functional airway organoids from iPSCs through distal (NKX2-1-positive) progenitors [67], CD47 was identified here among the genes with increasing expressions towards a lung phenotype. During (airway) epithelial maturation [68] and even during natural aging processes CD47 levels were found to be increased, concurrent with a reduction of self-renewal transcription factors, an observation that correlated with (age-related) tissue dysfunctions [69]. Signaling via the thrombospondin-1 (THSB1) receptor CD47 is generally known to limit the survival of cells and tissues under stress, which is due to limitations in cellular proliferation and also in asymmetric divisions [70]. Of note, CD47 signaling inhibition fostered epithelial cell recovery after injury through promotion of proliferation and self-renewal and thus provided a strong rationale for a potential therapeutically targeting of CD47 [71]. Accordingly, we found reduced expression levels of CD47 particularly in all alveolar (and also in the basal cell) clusters following radiation treatment, confirming on the hand the importance of this factor for the alveolar compartment. Reduced CD47 levels at the same time might further strengthen the regenerative capacity of respective cells, as increased portions of the alveolar #1 (AECI/II lineage-prone cells) and alveolar #3 (AECII) clusters could be revealed following RT.

### The FIB-specific RT response

Similar to the upregulation of GADD45G in alveolar epithelial cells after RT in alveolar epithelial cells, an upregulation of GADD45A in the fibroblasts subsets was observed. Mechanistically GADD45A can contribute to p38 activation either by activation of upstream kinase(s) or by direct interaction finally mediating DNA damage, cell cycle checkpoint control, and growth arrest [72]. According to the reported role of GADD45A in G2/M arrest [72, 73], the fibroblasts of our LuOrgs (mainly FIB#1) showed a G2/M arrest following RT together with an altered potentially activated phenotype. Increased TRIAP1 expression levels for example were observed here following RT. TRIAP1 is a well-known fibroblastic protein involved in mediating radiation resistance of (malignant) epithelial cells [74, 75]. Increased TRIAP1 expression and secretion by activated fibroblasts following RT was shown to mediate this resistance, further suggesting that blocking TRIAP1 activity and thus avoiding drug resistance may offer a promising drug development strategy [76]. Irradiation is generally known to trigger a strong (pro-fibrotic) response in different fibroblast subtypes, especially myofibroblasts finally leading to fibroblast activation while cell death is avoided [77, 78]. This RT-induced fibroblast fate included permanent senescent state, which in turn impacts on neighboring cells and the microenvironment itself, i.e. regulating extracellular matrix turnover by matrix metalloproteinases-2 (MMP-2) [79, 80], impacting on epithelial apoptosis by the aspartic protease cathepsin D (CTSD) [81], and modulating inflammation by APOBEC3C [82], all fibroblastic genes upregulated here following RT. Among the SASP factors also THSB1 could be detected with increased levels in the FIB #1 cluster representing senescent alveolar fibroblasts following RT. Fibroblast derived THSB1 is known to exhibit senescence-promoting activity towards neighboring cells, an effect that is at least partly related to THSB1-dependent activation and further ROS- and p38 MAPK-related activity of the profibrotic cytokine TGF-β1 [83]. Similarly, accumulation of this SASP factor in the extracellular matrix turned out to be a significant contributor to the stress response observed during aging process and mediating age-related diseases [84], and thus provides a strong rationale for a potential therapeutically targeting of CD47. Particularly concerning radiation-induced damage of normal tissues the THSB1-CD47-axis gained attraction as targeting either one of both factors exhibits the potential of radioprotection while preferring radio sensitization of tumors [85, 86].

## Conclusion

The use of disease-specific organoids has facilitated the analysis of underlying molecular mechanisms and successfully paved the way to personalized medicine through the identification of potentially new biomarkers and the development of patient-specific platforms for toxicological studies. We used a quite simple and thus very practicable but reliable lung organoid model without the need of using a matrix, that would be a step forward towards animal-origin and/or component-free in vitro modelling. According to the general limitation of lung organoids, that is inability to fully recapitulate the naïve lung cellular composition particularly concerning e.g. endothelial and immune cells, our LuOrgs also do not contain immune cell subsets. But for a patient-oriented screening platform, this may not be necessary (yet), if important insights into cell fate (e.g. cell death versus senescence) can be obtained during irradiation - as shown here - or if radiation sensitivities of resident cell types are determined and then investigated with regard to regeneration and therapeutic modulation. The presented results regarding our LuOrg model demonstrate that many features of RILI observed in human patients can be reproduced in vitro, representing a useful preclinical model for deciphering the mechanisms of radiation injury for identifying potential biomarkers, and drug targets of RILI progression. Accordingly, we provide here new candidate gene lists, which can be used to identify and specify a general and a cell-type dependent RT response in the lung, particular concerning senescence.

Thus, the enormous effort that has been put into developing 3D models as experimental tools successfully yielded more natural in vivo-like culture conditions such as the presence of ECM and even appropriate spatial and signaling interactions with other tissue-specific cells that in turn increase the translational relevance of respective studies. Another step forward towards the potential best in vitro culture systems of the normal in vivo situation might be represented by tissue slicing. So-called precision cut lung slices emerged as intermediary between cellular and organ-based studies, particularly for respiratory research [87, 88]. These living tissue preparations maintain their intercellular interactions among all resident cells, including the different epithelial cells, fibroblasts, and vascular cells, as well as cell-to-matrix relationships within the complex structures of the lung; and are therefore likely to bridge the gap between cell culture and a living organism.

## Electronic Supplementary Material

Below is the link to the electronic supplementary material.

Supplementary Document S1 including Figures S1–S5 and Table S1-S2

## Data Availability

The RNA-seq data (bulk and single cell) have been deposited at Gene Expression Omnibus (GEO) and are publicly available as of the date of publication (accession number: GSE275539). Any additional information required to reanalyze the data reported in this paper is available from the corresponding author upon request.

## Declarations

### Artificial Intelligence (AI)

The authors declare that they have not use AI-generated work in this manuscript.

## Abbreviations

iPSC: Induced pluripotent stem cell
LuOrg: Lung organoid
RILI: Radiation-induced lung injury
RT: Radiotherapy
SASP: Senescence associated secretory phenotype
XRT: (Photon) radiotherapy
