# Supplementary material for: Matrix-free human lung organoids derived from induced pluripotent stem cells to model lung injury
Source: Stem Cell Res Ther. 2024 Dec 18;15:468. doi: 10.1186/s13287-024-04106-3 (PMC11657174; doi:10.1186/s13287-024-04106-3)
Supplement: Supplementary file 1 — Supplementary Document S1 including Figures S1–S5 and Table S1-S2 [file 13287_2024_4106_MOESM1_ESM.pdf]

Supplemental Information

Matrix-free Human Lung Organoids Derived From Induced Pluripotent Stem Cells To Model Lung Injury

Bettina Budeus, Chiara Kroepel, Lisa Marie Stasch, Diana Klein

Supplemental Figures and Legends

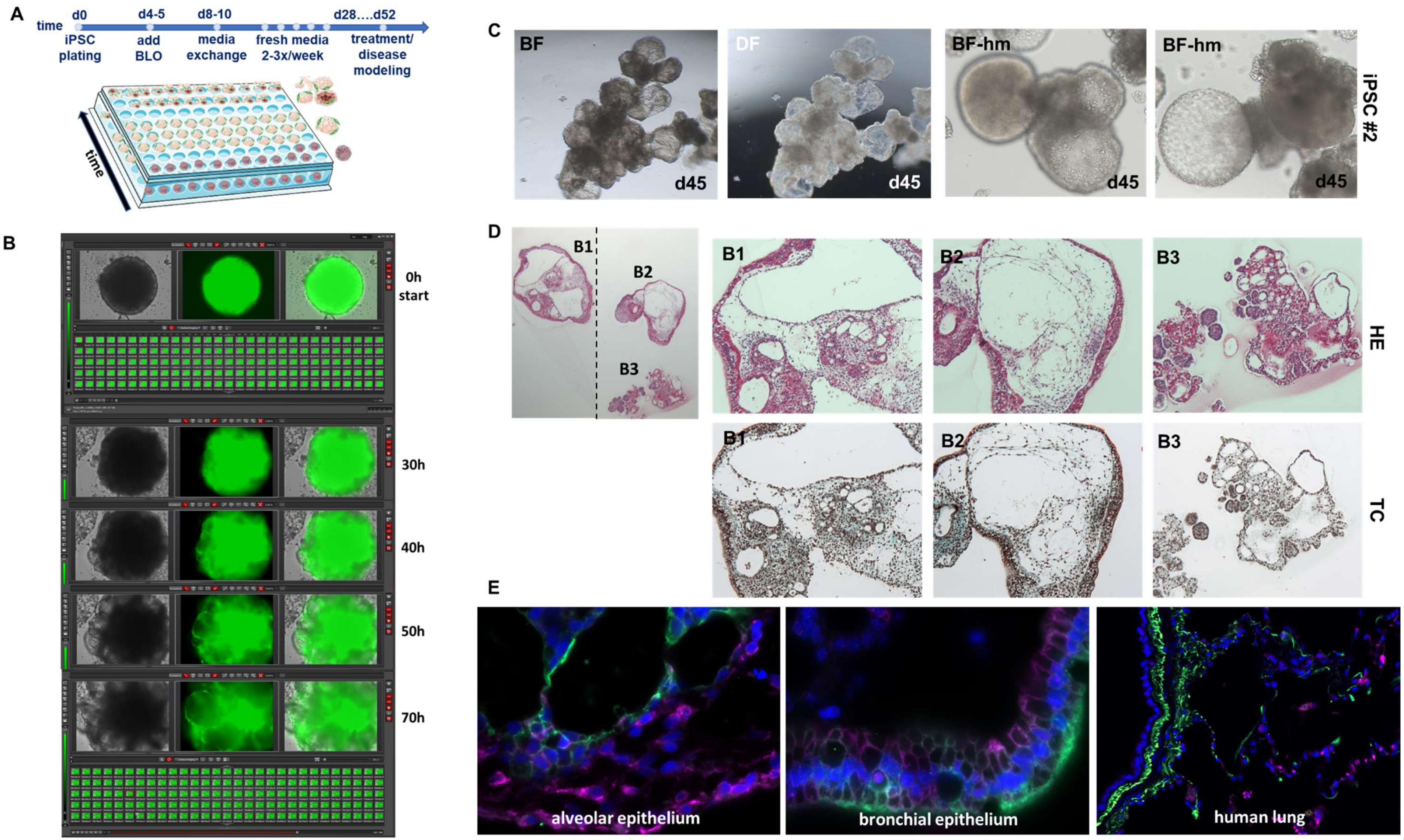

Figure S1. Characterization of free-floating iPSC-derived human LuOrgs, related to Figure 1.

(**A**) Scheme for the experimental design including the time line. (Created using Servier Medical Art; licensed under CC BY 4.0). (**B**) Continuous live cell imaging of GFP-transduced iPSCs (for better visualization) to track the lung structures forming. iPSCs were cultured as EBs in ULA-plates for 4-5 days prior BLOB medium addition. After another 4-5 days, medium was exchanged and recording started (d0) Representative screenshots are shown. (**C**) Bright-field (BF) and dark-field (DF) images of LuOrgs generated matrix-free from human iPSCs in ultra-low attachment plates at indicated time points using the 5x and 20x objective of an inverted microscope. (**D**) Histology staining of paraffin-embedded sections of the lung-like regions of LuOrgs using trichrome (TC) and hematoxylin and eosin (HE) staining. B1-B3 represent different LuOrg structures within the same section. Normal human lung tissue was included as positive control. Magnification: 5x. (**E**) Immunofluorescence staining of paraffin-embedded sections of LuOrgs representing different the lung-like regions for the lung epithelial markers ECAD (green). Nuclei were counterstained using DAPI (blue).

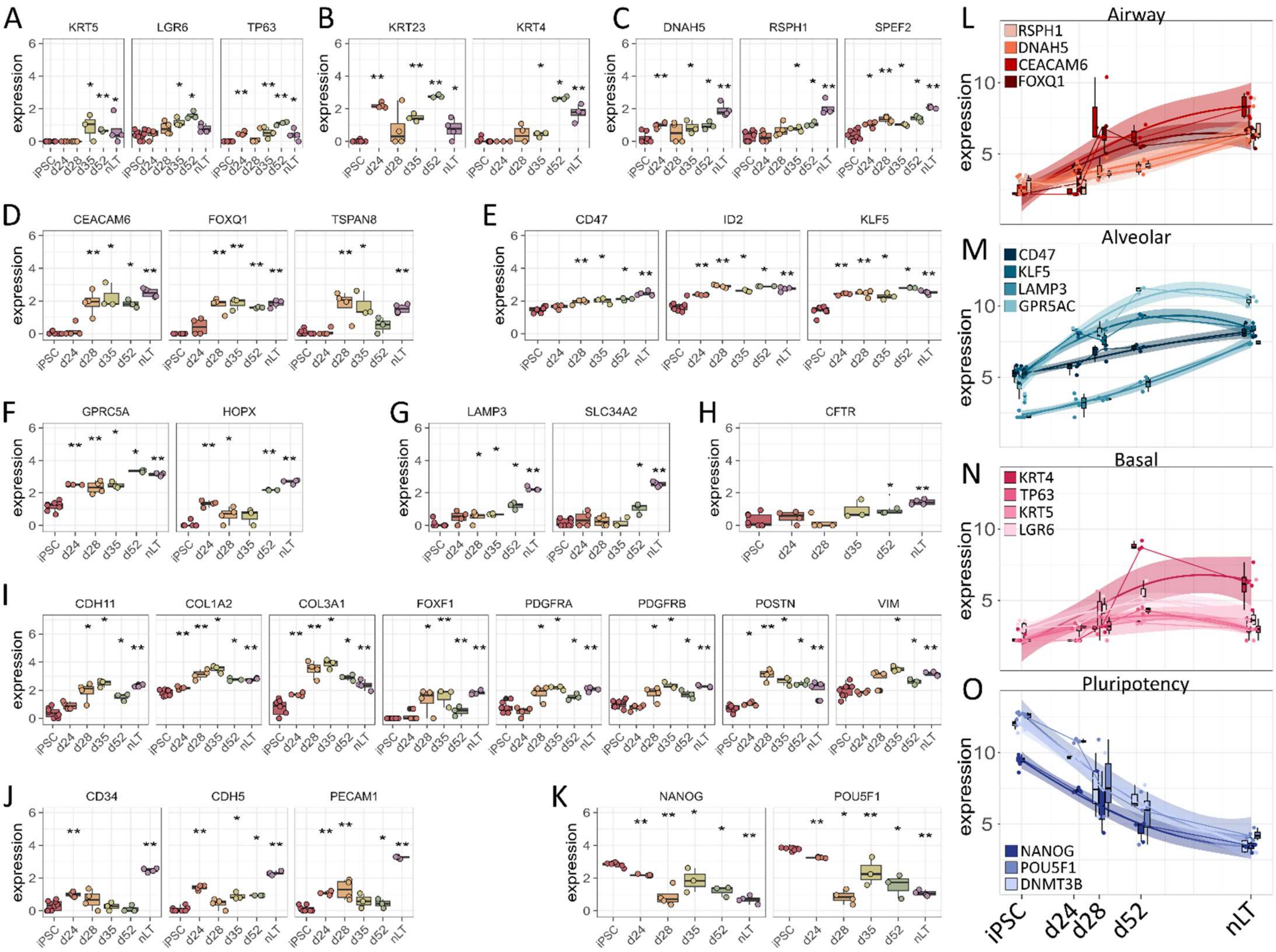

Figure S2. Molecular differentiation of six time points of generated lung cells, related to Figure 2.

(A - K) Normalized expression of iPSCs (8 samples), generated lung organoids at d24 (4 samples), d28 (4 samples), d35 (3 samples), d52 (3 samples) and nLT (4 samples) signature genes for (A) basal, (B) suprabasal, (C) ciliated, (D) goblet, (E) alveolar, (F) AECI, (G) AECII, (H) ionocyte, (I) fibroblastic, (J) endothelial and (K) pluripotent cells. (L-O) Normalized expression of selected genes categorized as (L) airway, (M) alveolar, (N) basal, and (O) pluripotency in iPSCs, d24, d28, d52 and nLT. Trend is calculated by Local Polynomial Regression Fitting (Loess). Mann-Whitney U test. * = 0.05, ** = 0.01, *** = 0.001. Shown are only comparisons between iPSCs and other timepoints.

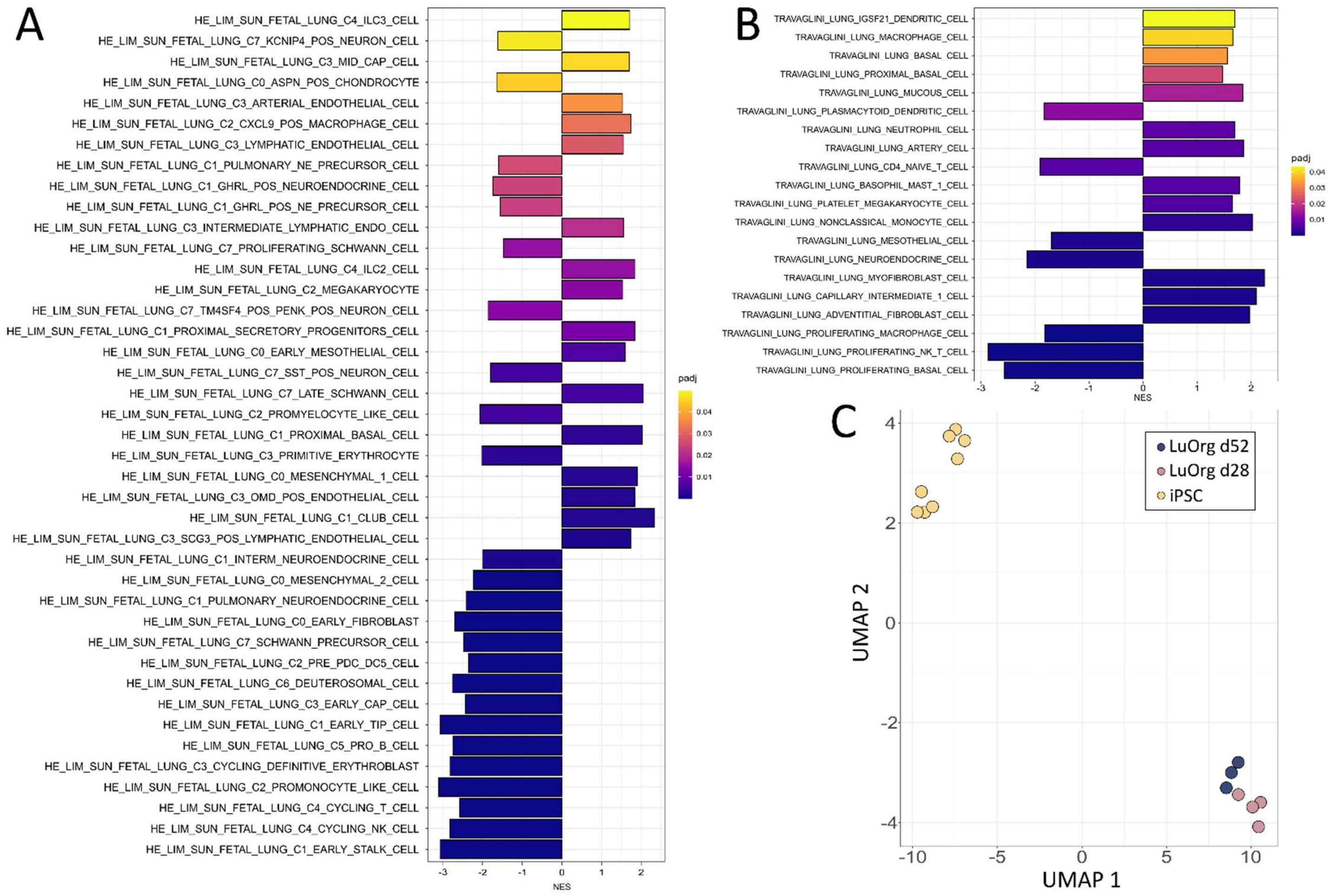

Figure S3. Lung cell-type dependent molecular characteristics of free-floating iPSC-derived human LuOrgs, related to Figure 2.

Normalized expression scores (x-axis) and adjusted p-values (color) of two marker gene sets, (**A**) the HE_LIM_SUN [12] and (**B**) the TRAVAGLINI [11] lung cell-type gene sets for significantly upregulated and downregulated gene sets using the bulk RNAseq data sets of generated LuOrgs (d52) and donor cells (iPSCs) depicted in Fig. 2A. (**C**) Uniform Manifold Approximation and Projection for Dimension Reduction (UMAP) representation of two batches of iPSCs (each four), three d28 and three d52 lung organoids using all expressed genes in all samples.

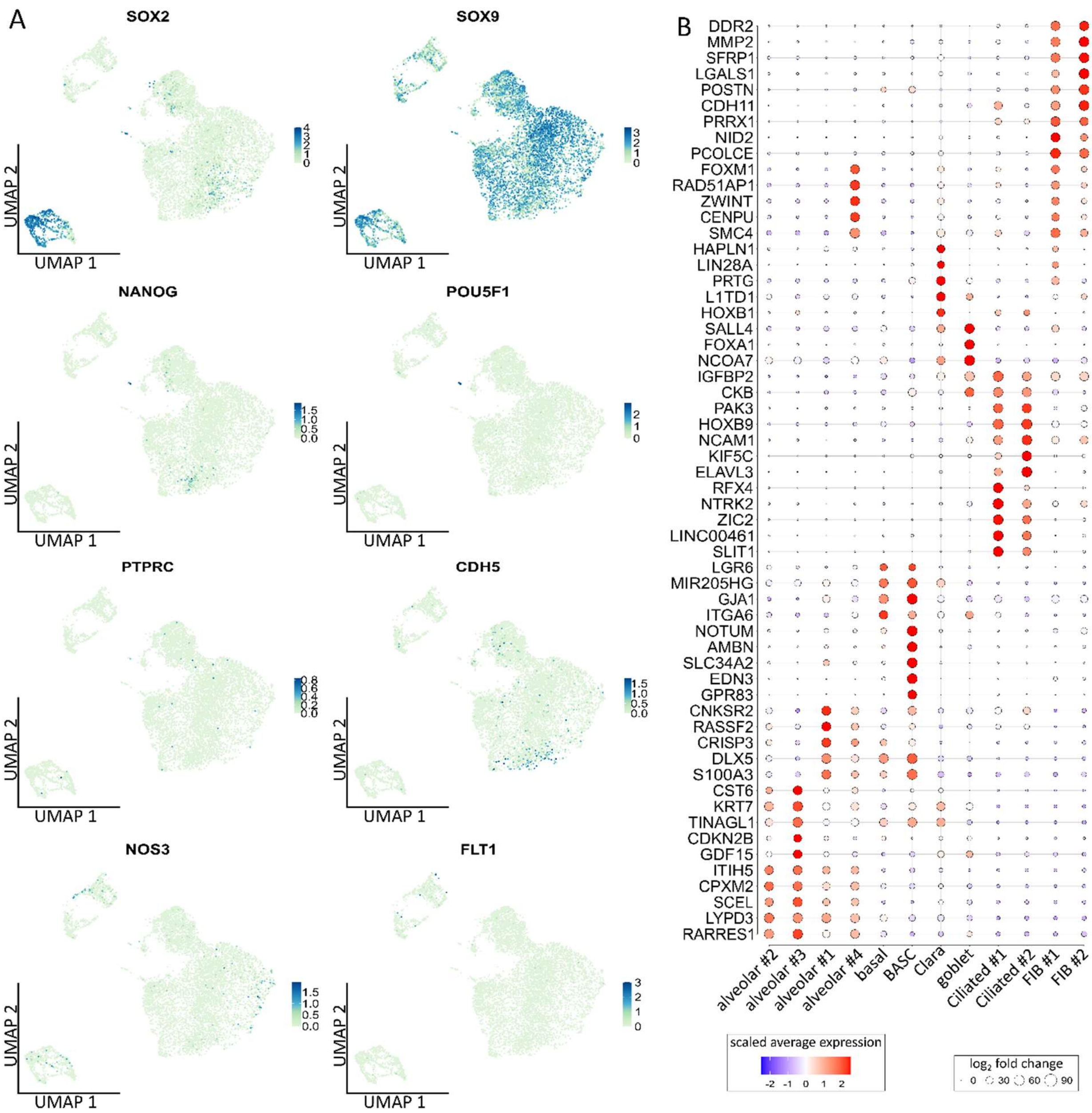

Supplemental Figure S4: Single cell analysis of the composition of lung organoids, related to Figure 3.

(**A**) Expression map of SOX2, SOX9, NANOG, POU5F1, PTPRC, CDH5, NOS3, and FLT1 in all clusters (N=3 biological replicates). (**B**) Dot plot of top five differentially expressed genes per cluster. Size of the dots indicate the percentage of cells in which this gene was found, the color indicates the normalized value of the expression.

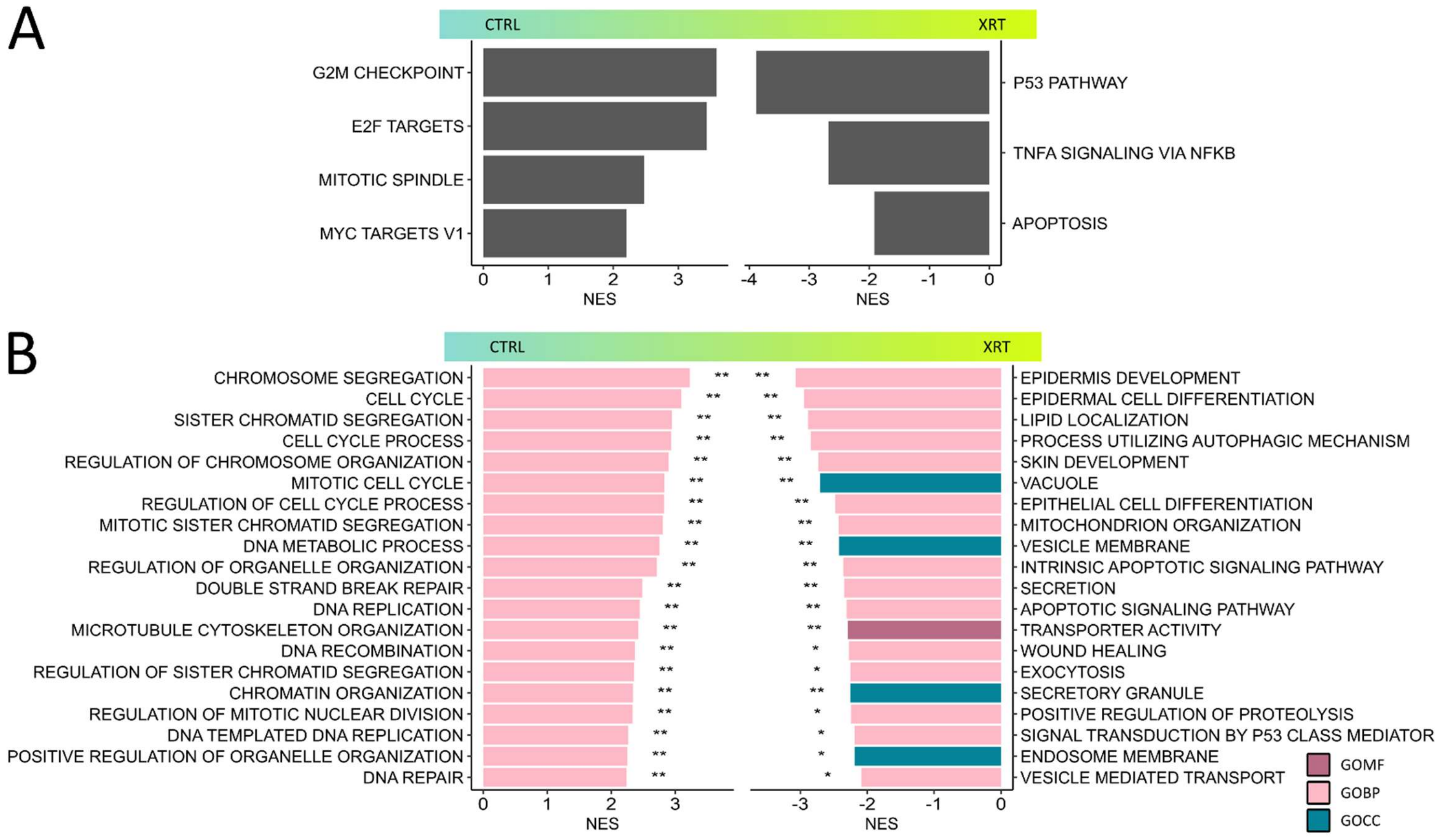

Supplemental Figure S5. Molecular characterization of response of LuOrgs to RT, related to Figure 4.

Significantly enriched Hallmark (**A**) and significantly enriched C5 ontology (**B**) gene sets showing normalized enrichment scores (NES) for significantly upregulated and downregulated gene sets during RT (bulk RNAseq). BP, biological process; CC, cellular component; MF, molecular function. P-values are calculated by adaptive multi-level split Monte-Carlo scheme (fgsea R-package)

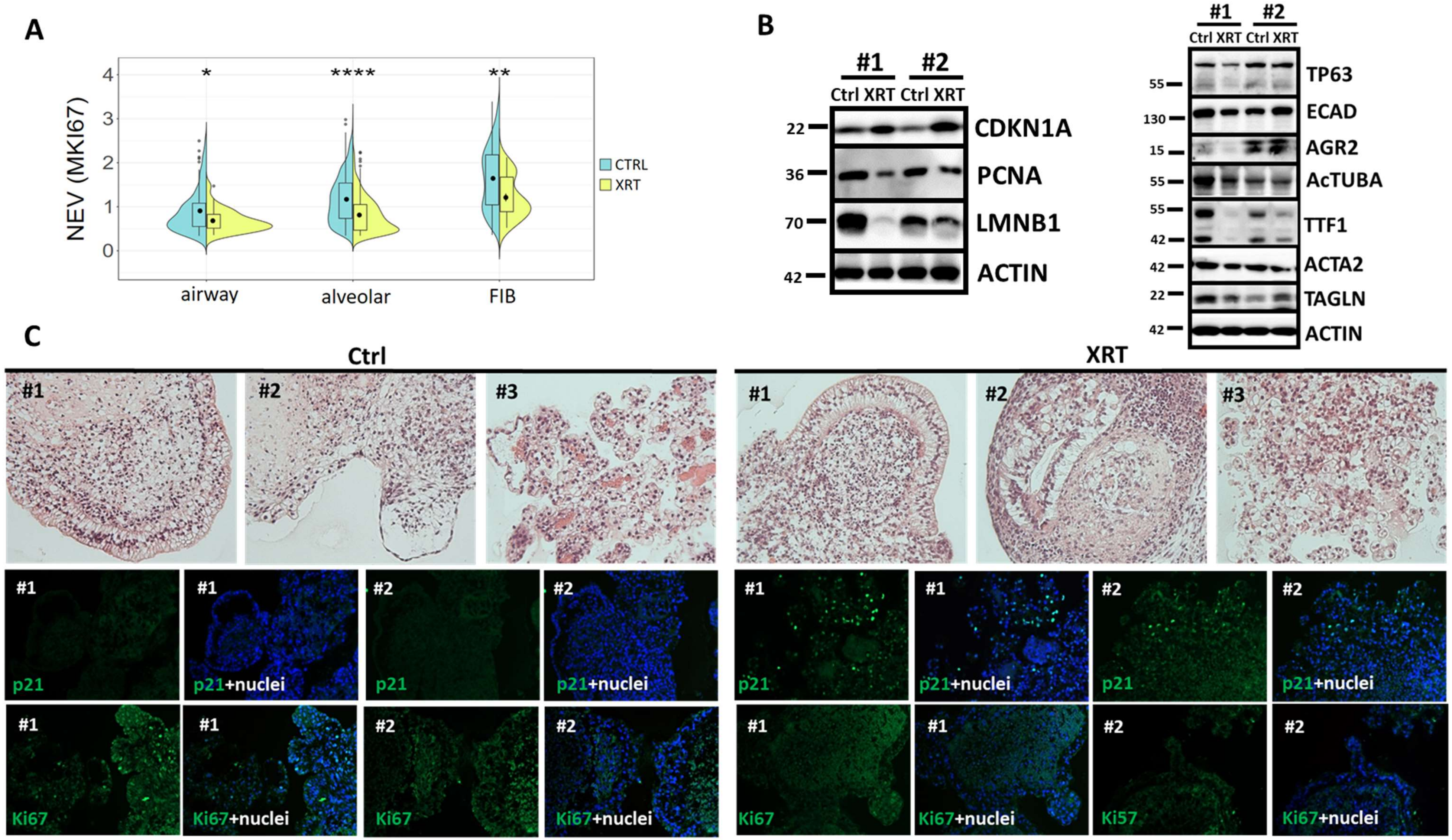

Supplemental Figure S6. Senescence induction in LuOrgs in response to RT, related to Figure 4-7.

(**A**) Normalized expression values as violin plots of MKI67 that is associated with cellular proliferation and ribosomal RNA transcription of each cluster before (CTRL) and after irradiation (XRT) for each cells expressing the marker. (**B**) Expression levels of the indicated proteins (senescence as well as

lung cell type markers) were analyzed in whole protein lysates of cultured LuOrgs (96 hours after XRT with 10Gy). Beta-Actin was included as loading control. Molecular weight markers are indicated. Representative blots are shown. (**D**) HE histology staining of paraffin-embedded sections of LuOrgs following radiation treatment. Magnification: 20x. (**E**) Immunofluorescence staining of paraffin-embedded sections of LuOrgs representing different the lung-like regions for the senescence marker CDKN1A (green) and the proliferation marker MKI67 (green). # numbers indicate different biological replicates. Nuclei were counterstained using DAPI (blue). Magnification: 10x.

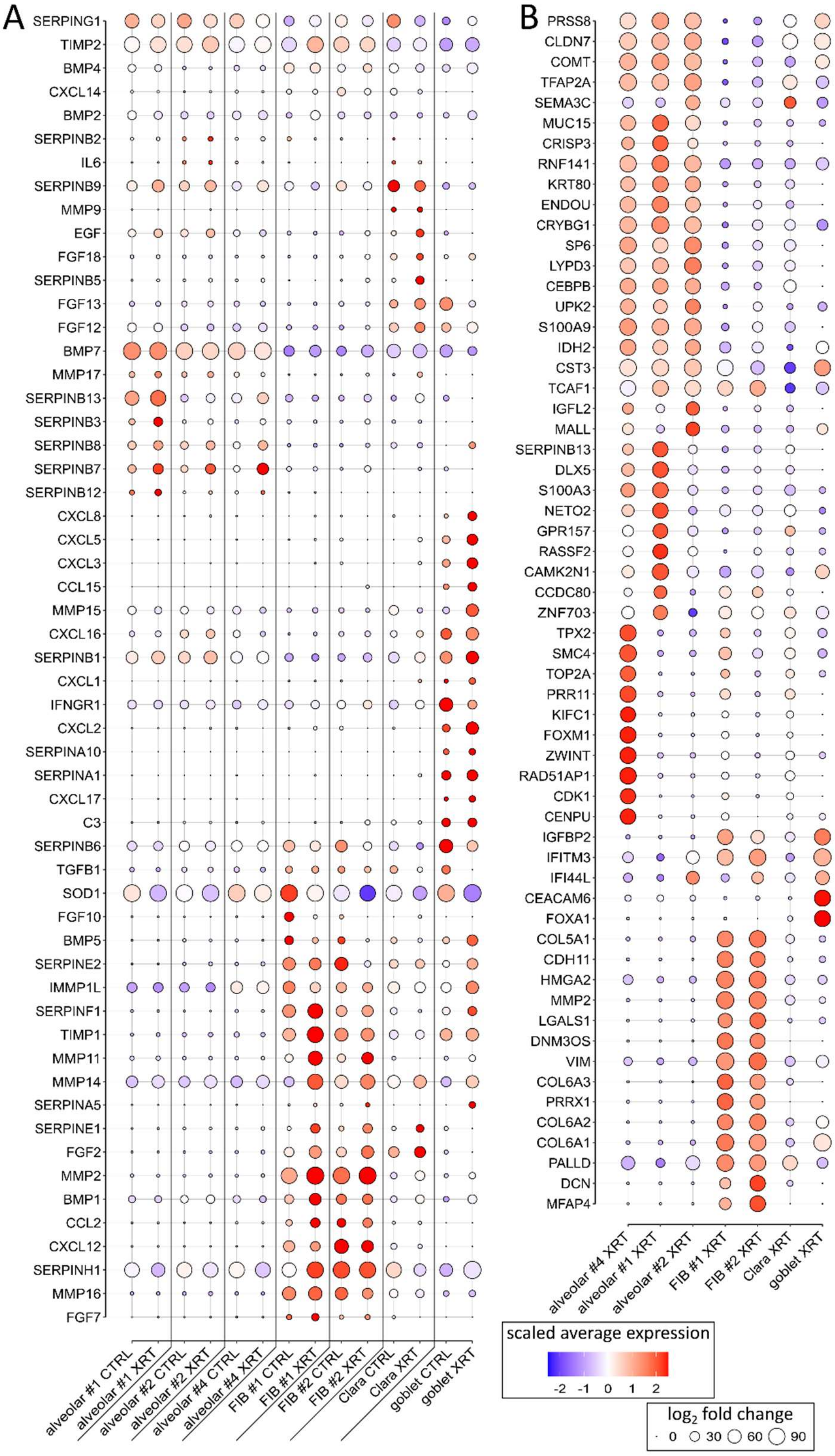

Supplemental Figure S7. Senescence-associated secretory phenotype (SASP) factor expression of LuOrgs in response to RT, related to Figure 6.

(**A**) Expression of known SASP genes in the clusters in which cells potentially became senescent following RT. (**B**) Dot plot of the top five differentially expressed genes per cluster after irradiation. Size of the dots indicate the percentage of cells in which this gene was found, the color indicates the normalized value of the expression..

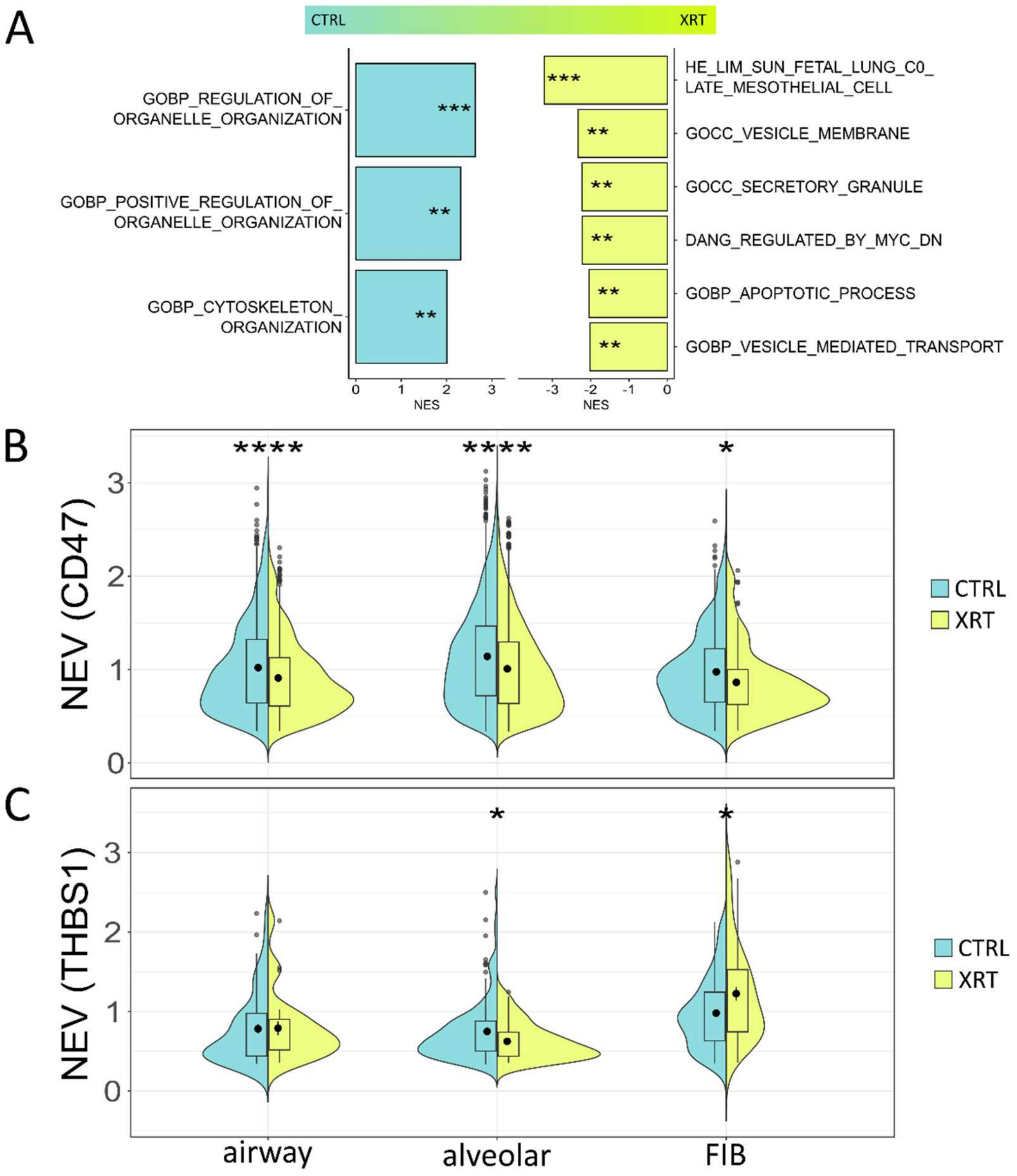

Supplemental Figure S8. The CD47-THBS1-axis of LuOrgs in response to RT, related to Figure 7.

(**A**) Significantly enriched gene sets containing CD47 showing normalized enrichment scores (NES) for significantly upregulated and downregulated gene sets (bulk RNAseq). Normalized expression values as violin plots of (**B**) CD47 and (**C**) THBS1 of each cluster before (CTRL) and after irradiation (XRT) for each cells expressing the marker.

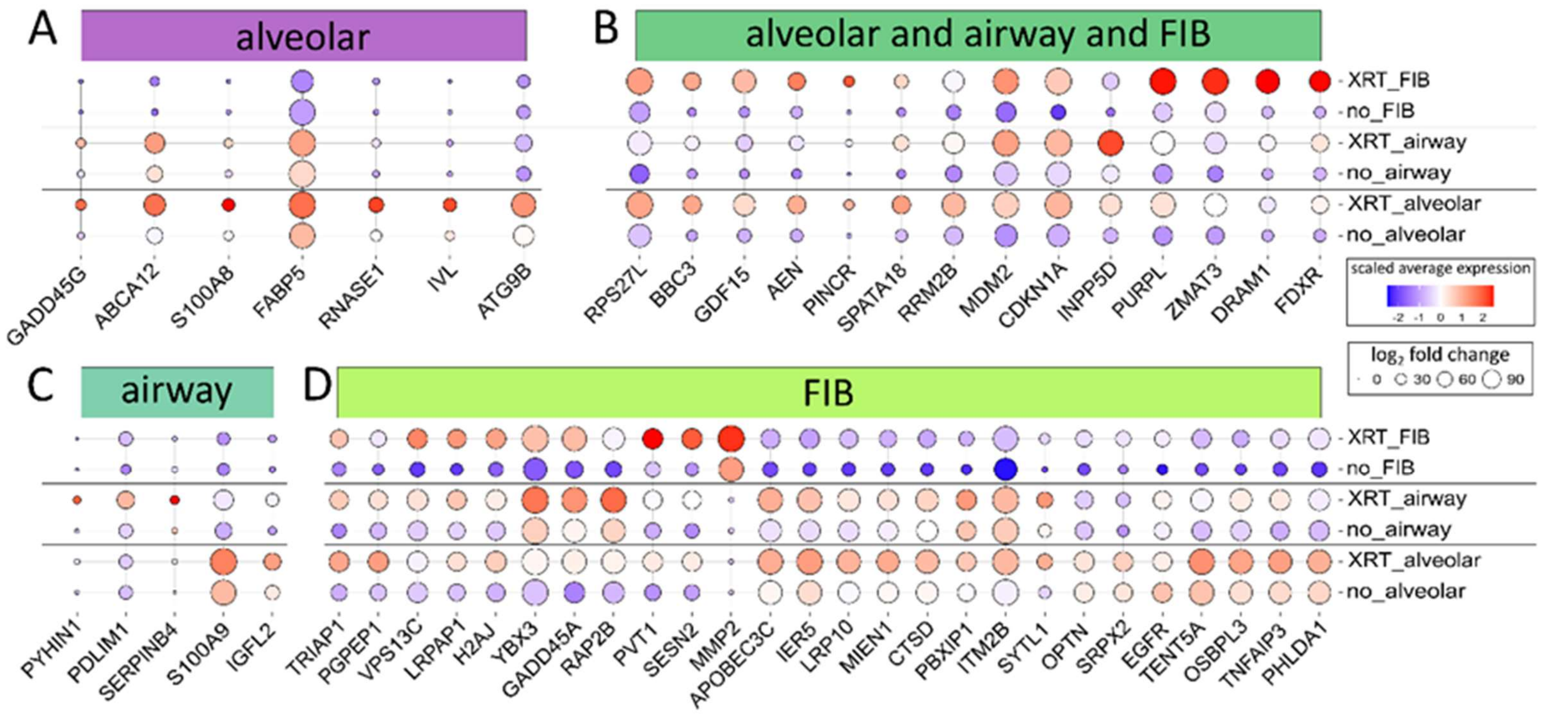

Supplemental Figure S9. Expression of significant genes in alveolar, airway and FIB meta cluster, related to Figure 7.

Dot plot of the top differentially expressed genes per meta cluster before and after irradiation. Size of the dots indicate the percentage of cells in which this gene was found, the color indicates the normalized value of the expression.

Table S1. RT-induced genes in lung organoid meta clusters, related to Figure 7.

Genes differentially expressed following RT in alveolar, airway, and FIB meta clusters as confirmed in the bulk RNAseq data sets.

1_2down	_2_3down	1_2_3up	1_2up	1_3up	1down	1up	2_3down	2down	3down	3up
8	15	7	6	115	8	41	5	5	28	51
VDR86-AS1	CDKN1A	CENPF	STC1	MKI67	IVL	HELLS	PHLDA3	IGFL2	SESN2	SFRP1
H2AC6	MDM2	SMC4	LGR6	TPX2	ABCA12	MYBL2	BLOC1S2	S100A9	GADD45A	RFLNB
CEL	BBC3	MCM6	VIM	PRR11	RNASE1	MSH6	DDB2	PDLIM1	SRPX2	IRX3
CDKN2B	GDF15	CDT1	COL4A2	ATAD2	S100A8	H1-3	PHPT1	PYHIN1	PHLDA1	RRM1
SERPINB3	RPS27L	RAD51AP1	ITGA9	H4C3	FABP5	POC1A	PPM1D	SERPINB4	OSBPL3	HMGB3
KRTAP19-1	PINCR	MCM5	HIF3A	ASPM	ATG9B	CDC25B			PGPEP1	FADS1
	ZMAT3			LMNB1	GADD45G	DBF4			YBX3	SMC1A
	AEN			MCM3		CEP135			ITM2B	RANBP1
	RRM2B			NASP		TK1			MIEN1	DPYSL2
	PURPL			TOP2A		SPAG5			SYTL1	VRK1
	SPATA18			E2F1		TACC3			TENT5A	TUBB2B
	FDXR			AURKB		FBXO17			TNFAIP3	SEPTIN6
	INPP5D			LDHA		PHF19			IER5	TPI1
	DRAM1			GINS2		SPON1			RAP2B	MCM7
				CDK1		ODC1			LRPAP1	DEK
				CENPE		NEK2			H2AJ	H2AZ1
				MCM10		DSCC1			LRP10	DHCR24
				SMC2		MYB			APOBEC3C	NCAPD2
				TMSB15A		MYOZ3			EGFR	NR2F1
				CLSPN		CHTF18			PBXIP1	DHFR
				KIF15		SCCPDH			OPTN	SMAD9
				KIFC1		PRC1			PVT1	HMGN2
				MCM4		CDC45			CTSD	CHCHD2
				DLGAP5		CYB5R2			VPS13C	PKM
				UBE2C		ECT2			TRIAP1	ERG
				NCAPH		CEP55			MMP2	USP1
				DTL		MNS1				HAUS1
				CKS1B		RECQL4				FRZB
				AURKA		BARD1				GPI
				PCLAF		CCDC18				AMOT
				RRM2		INCENP				EZH2
				HMGB2		SUV39H1				GJC1
				KIF20B		USP13				EMP2
				KIF20A		TCF7				ENO1
				MAD2L1		POLQ				KLHL23
				CCNB1		HROB				SCUBE1
				CDCA8		CD320				MTHFD1
				CDC6						
				CDC20						
				CHAF1A						
				CIP2A						
				PTTG1						
				CDCA7						
				UHRF1						
				TMEM97						
				FXYD6						
				KIF2C						

				CCNA2						
				RFC3						
				KIF22						
				BIRC5						
				ANLN						
				FANCD2						
				ARHGAP11A						
				GMNN						
				TROAP						
				BRCA1						
				CCNB2						
				CENPU						
				HJURP						
				ZWINT						
				DIAPH3						
				CDCA5						
				SGO1						
				FANCI						
				CDCA3						
				DUT						
				HMMR						
				WDHD1						
				CDCA2						
				NCAPG2						
				BLM						
				CENPH						
				FEN1						
				NDC80						
				CENPJ						
				CDC25A						
				NCAPG						
				CKAP2L						

Table S2. Key resources table

REAGENT or RESOURCE	SOURCE	IDENTIFIER
Antibodies		
AQP5 Polyclonal antibody	Proteintech	Cat#20334-1-AP
SFTPC Polyclonal antibody	Proteintech	Cat#10774-1-AP
AGR2 Polyclonal antibody	Proteintech	Cat#66768-1-Ig
alpha-Smooth Muscle Actin Antibody (1A4/asm-1) APC	Biotechne Novus	Cat#NBP2-34522APC
CD324 (E-Cadherin) Monoclonal Antibody (DECMA-1), Alexa Fluor™ 488, eBioscience	ThermoFischer	Cat#53-3249-82
CD326 (EpCAM) Monoclonal Antibody (G8.8), PE, eBioscience™	ThermoFischer	Cat#12-5791-82
Vimentin Monoclonal Antibody (3H9D1), CoraLite® 594	ThermoFischer	Cat#CL594-60330
Podoplanin Monoclonal Antibody (NZ-1.3), APC, eBioscience™	ThermoFischer	Cat#17-9381-42
CoraLite® Plus 488-conjugated Caveolin-1 Monoclonal antibody QN:Q1604572	Proteintech	Cat#CL488-66067
CoraLite® Plus 488-conjugated acetylated Tubulin (Lys40) Monoclonal antibody	Proteintech	Cat#CL488-66200
CoraLite® Plus 488-conjugated p63 Polyclonal antibody	Proteintech	Cat#CL488-12143
CD34 Class II, Clone QBEnd 10	Agilent/ DAKO	Cat#M716501-2
Hoechst 33342	ThermoFischer	Cat#H1399
ALEXA FLUOR 555	ThermoFischer	Cat#A21424
ALEXA FLUOR 488	ThermoFischer	Cat#A11034
Biological samples		
Normal lung tissue (human)	This paper; Steens et al. [90]; Sentek et al. [89]	doi.org/10.1002/sctm.20-0191; 10.3389/fonc.2024.1406268
Chemicals, peptides, and recombinant proteins		
Hu Recom FGF-7 ACF	StemCell Technologies	Cat#78186.1
HURecom FGF-10(KGF-2) ACF	StemCell Technologies	Cat#78173.1
L-Ascorbic acid	Sigma-Aldrich	Cat#A92902
Monothioglycerol	Sigma-Aldrich	Cat#M6145
Bovine Serum Albumin	Roche	Cat#BSAVHS-RO
VitronectinXF	StemCell Technologies	Cat#100-0763
Laduviglusib (CHIR-99021)	Biozol/Selleckchem	Cat#S1263
C12FDG	Invitrogen	Cat#D2893
DMEM-F12	Invitrogen	Cat#11330-032
B-27™ Supplement (50x),	ThermoFischer/Gibco	Cat#17504044
N-2 Supplement (100x)	ThermoFischer/Gibco	Cat#17502048
Hydrocortisin Stock Solution	StemCell Technologies	Cat#7926
0,2% Heparin Solution	StemCell Technologies	Cat#7980
L-Ascorbic acid	Sigma-Aldrich	Cat#A92902
7-AAD	Invitogen	Cat#A1310
TrypLE	ThermoFischer	Cat#12604013
Normal goat serum	ThermoFischer	Cat#31873
Fetal calf serum	ThermoFischer/Gibco	Cat#A5256701

ATRA	StemCell Technologies	Cat#72262
Critical commercial assays		
Rhapsody cDNA Kit	BD	Cat#633773
Human Single-Cell Multiplexing Kit	BD	Cat#633781
BD Rhapsody WTA Amplification Kit	BD	Cat#633801
RNeasy Plus Mini Kit (50)	Qiagen	Cat#74134
QuantiTect Rev. Transcription Kit (50)	Qiagen	Cat#205311
qPCR 2X MasterMix for SYBR® Assay ROX	Eurogentec	Cat#05-SN2X-03T
Gentle Cell dissociation Reagent cGMP	StemCell Technologies	Cat#100-0485
CellAdhere Diltution Buffer	StemCell Technologies	Cat#7183
mTeSR1 Complete Kit GMP	StemCell Technologies	Cat#85850
Deposited data		
Raw and analyzed data (bulk RNA)	This paper	GEO: GSE275539
Raw and analyzed data (single cell)	This paper	GEO: (already uploaded, waiting for confirmation)
GSEA (of single cell gene sets in bulk RNA data sets	This paper	Table S1
Experimental models: Cell lines		
SCTi003 iPSC cell line (iPSC #1)	StemCell Technologies	Cat#200-0511
iPS01 iPSC cell line (iPSC #2)	ALSTEM Inc	Cat#iPS01
Oligonucleotides		
CDK1_fw GGAAACCAGGAAGCCTAGCATC	This paper / metabion	N/A
CDK1_rev GGATGATTCAGTGCCATTTTGCC	This paper / metabion	N/A
LMNB1_fw GAGAGCAACATGATGCCCAAGTG	This paper / metabion	N/A
LMNB1_rev GTTCTTCCCTGGCACTGTTGAC	This paper / metabion	N/A
CDKN1A_fw AGGTGGACCTGGAGACTCTCAG	This paper / metabion	N/A
CDKN1a_rev TCCTCTTGGAGAAGATCAGCCG	This paper / metabion	N/A
bACT_fw CACCATTGGCAATGAGCGGTTC	This paper / metabion	N/A
bACT_rev AGGTCTTTGCGGATGTCCACGT	This paper / metabion	N/A
Software and algorithms		
Hisat 2 v2.2.1	10.1101/gr.275193.120	http://daehwankimlab.github.io/hisat2/manual/
CytExpert Software	Beckman Coulter	https://beckman.de/en/flow-cytometry/research-flow-cytometers/cytoflex/cytoflex-startup-guide
TrimGalore v0.6.0	10.14806/ej.17.1.200	https://github.com/FelixKrueger/TrimGalore
R-package GenomicAlignments v1.40.0	10.1371/journal.pcbi.1003118	https://bioconductor.org/packages/release/bioc/html/GenomicAlignments.html
Cwl-runner v2.0		https://bitbucket.org/CRSwDev/cwl/src/master/

Seurat v4.0	10.1016/j.cell.2021.04.048	https://satijalab.org/seurat/
EnhancedVolcano v1.22.0	Blighe K, Rana S, Lewis M (2024). EnhancedVolcano: Publication-ready volcano plots with enhanced colouring and labeling. R package version 1.22.0, https://github.com/kevinblighe/EnhancedVolcano.	https://bioconductor.org/packages/release/bioc/html/EnhancedVolcano.html
ggalluvial v0.12.5	10.21105/joss.02017	https://cran.r-project.org/web/packages/ggalluvial/
nVennR v0.2.3	10.1093/bioinformatics/bty109	https://github.com/cran/nVennR
fgsea v1.30.0	10.1101/060012	https://bioconductor.org/packages/release/bioc/html/fgsea.html
SCpubr v2.0.0	10.1101/2022.02.28.482303	https://github.com/enblacar/SCpubr
ggplot2 v3.5.1	Wickham H (2016). ggplot2: Elegant Graphics for Data Analysis. Springer-Verlag New York. ISBN 978-3-319-24277-4	https://ggplot2.tidyverse.org/
Other		
BIOFLOAT 96-well plate	SARSTEDT	Cat#83.3925.400
